# Haploinsufficiency of *ITSN1* is associated with a substantial increased risk of Parkinson’s disease

**DOI:** 10.1016/j.celrep.2025.115355

**Published:** 2025-03-07

**Authors:** Thomas P. Spargo, Chloe F. Sands, Isabella R. Juan, Jonathan Mitchell, Vida Ravanmehr, Jessica C. Butts, Ruth B. De-Paula, Youngdoo Kim, Fengyuan Hu, Quanli Wang, Dimitrios Vitsios, Manik Garg, Lawrence Middleton, Michal Tyrlik, Mirko Messa, Guillermo del Angel, Daniel G. Calame, Hiba Saade, Laurie Robak, Ben Hollis, Vishnu A. Cuddapah, Huda Y. Zoghbi, Joshua M. Shulman, Slavé Petrovski, Ismael Al-Ramahi, Ioanna Tachmazidou, Ryan S. Dhindsa

**Affiliations:** 1 Centre for Genomics Research, Discovery Sciences, R&D, AstraZeneca, Cambridge, UK; 2 Department of Pathology and Immunology, Baylor College of Medicine, Houston, TX, USA; 3 Genetics & Genomics Graduate Program, Baylor College of Medicine, Houston, TX, USA; 4 Jan and Dan Duncan Neurological Research Institute, Texas Children’s Hospital, 1250 Moursund St., Suite N.1150, Houston, TX, USA; 5 Department of Molecular and Human Genetics, Baylor College of Medicine, Houston, TX, USA; 6 Department of Bioengineering, George R. Brown School of Engineering, Rice University, Houston, TX, USA; 7 Quantitative and Computational Biology Graduate Program, Baylor College of Medicine, Houston, TX, USA; 8 Centre for Genomics Research, Discovery Sciences, R&D, AstraZeneca, Waltham, MA, USA; 9 Translational Genomics, Centre for Genomics Research, Discovery Sciences BioPharmaceuticals R&D, AstraZeneca, Gothenburg, Sweden; 10 Department of Pediatrics, Texas Children’s Hospital, Houston, TX, USA; 11 Department of Neurology, Baylor College of Medicine, Houston, TX, USA; 12 Howard Hughes Medical Institute, Baylor College of Medicine, Houston, TX, USA; 13 Department of Neuroscience, Baylor College of Medicine, Houston, TX, USA; 14 Center for Alzheimer’s and Neurodegenerative Diseases, Baylor College of Medicine, Houston, TX, USA; 15 Department of Medicine, Austin Health, University of Melbourne, Melbourne, VIC, Australia; 16 These authors contributed equally; 17 Lead contact

## Abstract

Despite its significant heritability, the genetic basis of Parkinson’s disease (PD) remains incompletely understood. Here, in analyzing whole-genome sequence data from 3,809 PD cases and 247,101 controls in the UK Biobank, we discover that protein-truncating variants in *ITSN1* confer a substantially increased risk of PD (*p* = 6.1 × 10^−7^; odds ratio [95% confidence interval] = 10.5 [5.2, 21.3]). We replicate this association in three independent datasets totaling 8,407 cases and 413,432 controls (combined *p* = 4.5 × 10^−12^). Notably, *ITSN1* haploinsufficiency has also been associated with autism spectrum disorder, suggesting variable penetrance/expressivity. In *Drosophila*, we find that loss of the *ITSN1* ortholog *Dap160* exacerbates α-synuclein-induced neuronal toxicity and motor deficits, and *in vitro* assays further suggest a physical interaction between ITSN1 and α-synuclein. These results firmly establish *ITSN1* as a PD risk gene with an effect size exceeding previously established loci, implicate vesicular trafficking dysfunction in PD pathogenesis, and potentially open new avenues for therapeutic development.

## In brief

Spargo et al. analyzed whole-genome sequence data from ~900,000 individuals and found that protein-truncating variants in *ITSN1* confer a ~10-fold increased risk of Parkinson’s disease. Functional studies suggest that ITSN1 physically interacts with α-synuclein and its loss exacerbates α-synuclein toxicity.

## Graphical abstract

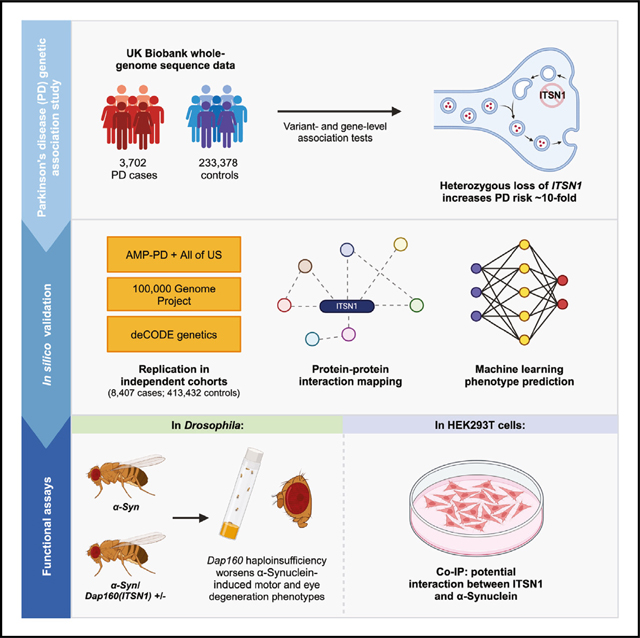

## INTRODUCTION

Parkinson’s disease (PD) is a heritable, age-related neurodegenerative disorder characterized by tremor at rest, rigidity, bradykinesia, loss of smell, and impaired gait/balance. Affecting 1%–2% of adults aged over 65 years, PD is the second most common neurodegenerative disease.^1^ Neuropathological hall-marks of PD include the presence of intracellular inclusions containing aggregates of fibrillated α-synuclein, known as Lewy bodies, and the degeneration of dopaminergic neurons in the substantia nigra pars compacta. Decreased dopamine release in the striatum and subsequent alterations in basal ganglia circuits are primarily responsible for PD motor manifestations.^2^ Current therapies provide symptomatic relief but are not curative, as they do not prevent dopaminergic neuron loss.

PD is thought to arise from a combination of environmental and genetic factors.^3^ Genetic discoveries in PD have uncovered key disease mechanisms, including mitochondrial and lysosomal dysfunction, impaired protein degradation,^4^ synaptic dysfunction, and disrupted vesicular trafficking. Initial studies focused on familial PD (5%–10% of cases), revealing rare, highly penetrant alleles in genes like *SNCA* (which encodes α-synuclein), *PRKN*, *GBA1*, and *LRRK2*. Genetic investigations of sporadic PD have primarily employed genome-wide association studies (GWASs), which have identified numerous loci, including some that map to familial PD risk genes.^5^ However, deriving biological insights from these common variants remains challenging due to their non-coding nature, small effect sizes, and vulnerability to confounding by linkage disequilibrium. Rare protein-coding variants, meanwhile, typically confer larger effect sizes and can therefore provide clearer mechanistic and therapeutic insights. Although there has been progress in identifying rare-variant associations in PD, the majority of PD heritability still remains unexplained.^3,6^

Here, we leveraged an unprecedented scale of whole-genome sequencing (WGS) data with paired health record data from ~500,000 UK Biobank (UKB) participants to expand our understanding of the genetic architecture of PD. Through variant- and gene-level analyses, we uncovered a loss-of-function signal in *ITSN1*, which encodes a protein involved in synaptic vesicle recycling. This work commenced independently of another recent study that identified an association between *ITSN1* and PD.^7^ We replicated this association in three additional case-control cohorts and performed *in vivo* and *in vitro* functional validation. This study adds to the growing evidence that vesicle trafficking dysfunction is a key driver in PD pathogenesis.

## RESULTS

### Cohort characteristics and study design

**Figure 1.**
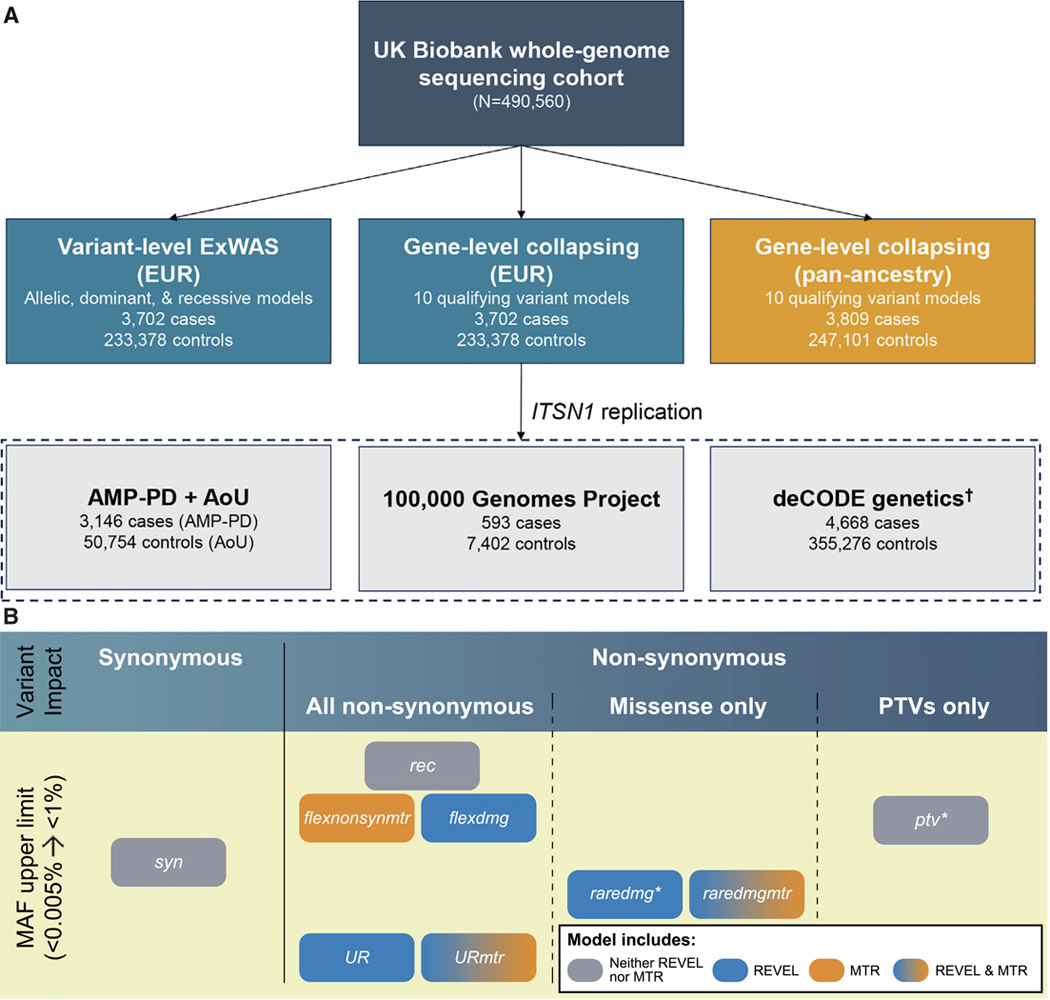
Genetic association study design (A) Schematic of the genetic association analyses. The discovery cohort included whole-genome sequenced participants from the UKB. The pan-ancestry analysis included individuals of European (EUR), African, East Asian, and South Asian genetic ancestries. The dotted box indicates the three additional EUR cohorts used to replicate the association between *ITSN1* and PD. ^†^The deCODE replication was performed using summary statistics from a recently published PD collapsing analysis.^7^ (B) Schematic of gene-level collapsing models. Qualifying variants were defined based on three major criteria: minor allele frequency (MAF) upper limit, variant impact, and missense scores (see Table S3). The *ptvraredmg* model, which combines the *ptv* and *raredmg* models, is not depicted. ExWAS, exome-wide association study; AMP-PD, Accelerating Medicines Partnership program for Parkinson’s Disease; AoU, All of Us.

We processed genome sequence data from 490,560 multi-ancestry UKB participants through our previously described cloud-based pipeline,^8^ removing samples with low sequencing quality or low coverage depth, and index-sample pruning among closely related individuals (see STAR Methods). We then identified individuals with PD by aggregating self-reported data, hospital record-derived billing codes, and death registries (Table S1). We grouped participants into genetic ancestry clusters based on genetic similarity, estimated through principal-component analysis. This approach mitigates confounding due to population stratification. However, we emphasize that genetic ancestry represents a continuum, and our classifications do not imply the existence of discrete or biologically distinct ancestral populations. In total, there were 3,809 PD cases: 3,702 predominantly of European, 69 predominantly of South Asian, 32 predominantly of African, and six predominantly of East Asian genetic ancestry (Figure 1A).

In European cases, the median age of PD diagnosis was 70 years (interquartile range [IQR], 63–75), and 2,331 (63%) were male, consistent with epidemiological observations that PD is twice as prevalent in men.^9^ To reduce potential contamination from genetically related diagnoses, we restricted controls to participants without any diagnosis under International Classification of Diseases, Tenth Revision (ICD-10) Chapter VI (diseases of the nervous system).^8^ We then downsampled the remaining controls to match the 2:1 sex ratio observed in cases, resulting in 233,378 controls (Table S2).

To test for protein-coding associations with PD, we performed variant-level exome-wide association studies (ExWASs) and gene-level collapsing analyses.^8^ We carried out two versions of collapsing analysis: one restricted to individuals of non-Finnish European (EUR) ancestry (>90% of the UKB cohort) and one across all four genetic ancestry groups in the UKB. Associations were considered study-wide significant at *p* ≤ 1 × 10^−8^ and suggestive at 1 × 10^−8^ < *p* ≤ 1 × 10^−6^ (see STAR Methods).

We performed gene-based replication analyses in three additional EUR cohorts (Figure 1A). One comprised 3,739 cases from the Accelerating Medicines Partnership program for Parkinson’s disease (AMP-PD) and 50,754 controls from All of Us (AoU). The second included 593 cases and 7,402 controls from the 100,000 Genomes Project (100kGP). The third cohort included 4,668 cases and 355,276 controls from a recently published rare-variant PD study in the Icelandic deCODE genetics cohort.^7^

### Variant-level associations

**Figure 2.**
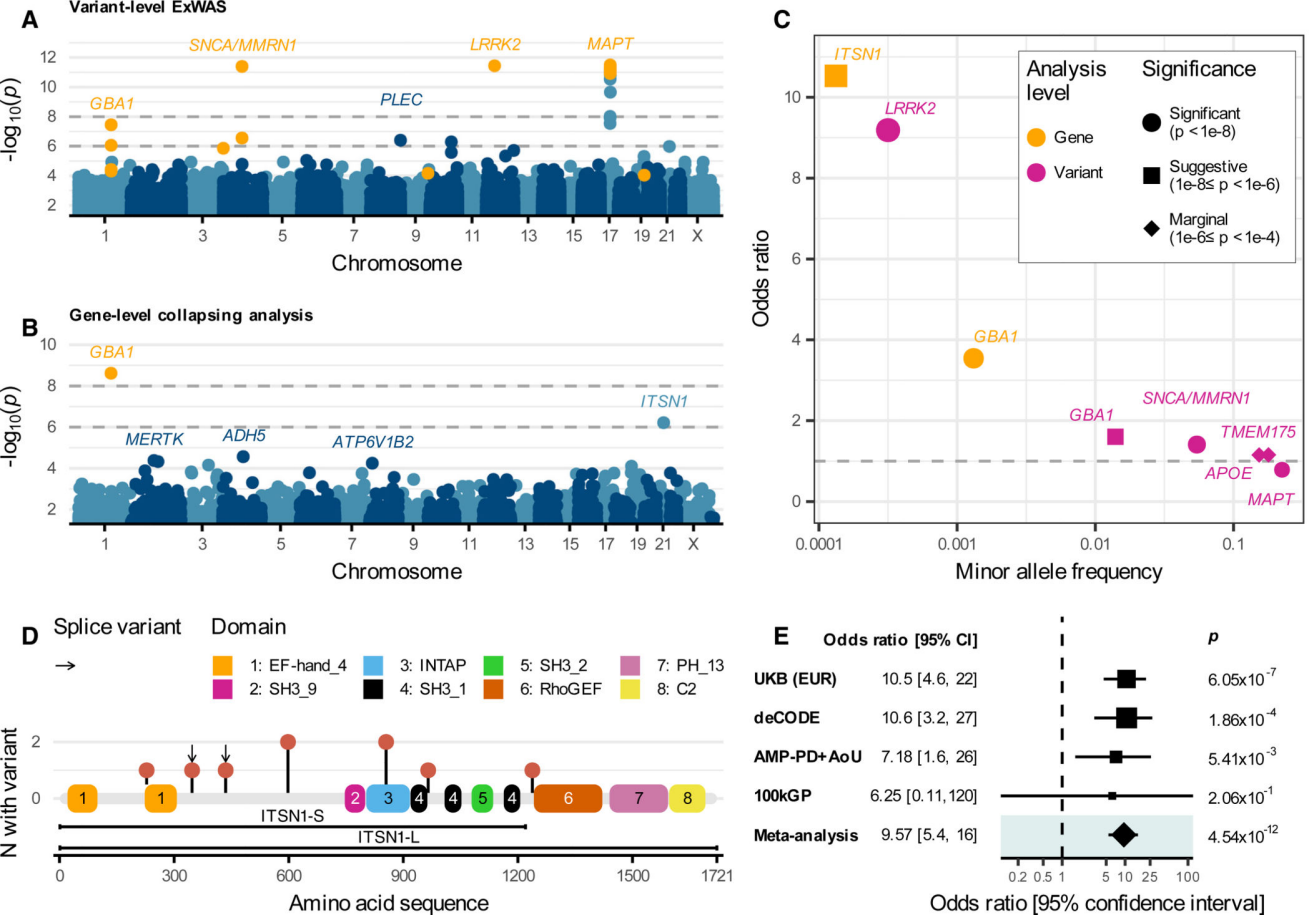
Variant- and gene-level associations with PD (A and B) Manhattan plots of variant-level (A) and gene-level (B) associations with PD in UKB Europeans. If the same association was detected in multiple models, we depicted the most significant association. Horizontal dashed lines indicate the significance (*p* <1 × 10^−8^) and suggestive (*p* <1 × 10^−6^) thresholds. Orange points indicate marginally significant (*p* < 1 × 10^−4^) associations in established PD genes. (C) Odds ratio versus MAFs for *ITSN1* and other established PD loci that achieved *p* < 1 × 10^−4^ in the ExWAS or collapsing analysis. (D) Lollipop plot showing the location of *ITSN1* protein-truncating variants in cases against the MANE transcript (ENST00000381318) and PFam domains of the corresponding protein (Q15811); bars below the transcript indicate the long and short isoforms. (E) Association between *ITSN1* PTVs and PD across the discovery and replication cohorts. Data are represented as odds ratios and 95% confidence intervals. *p* values in individual cohorts were generated via a two-tailed Fisher’s exact test; the combined *p* value was calculated via an exact Cochran-Mantel-Haenszel (CMH) test. EUR, European-ancestry cohort; AMP-PD, Accelerating Medicines Partnership program for Parkinson’s Disease; AoU, All of Us; 100kGP = 100,000 Genomes Project. See also Tables S3–S5; Figures S1–S4.

We first performed a variant-level ExWAS in the UKB EUR cohort to identify protein-coding variants associated with PD risk. Genomic inflation was well-controlled across the tested additive, dominant, and recessive genetic models (Figure S1; λ_GC_ range, 1.01, 1.02). There were 36 significant (*p* ≤ 1 × 10^−8^) variants across three independent loci (Table 1; Table S3). These results confirmed previously established associations, including the p.Gly2019Ser *LRRK2* variant (*p* = 3.6 × 10^−12^; odds ratio [OR]; 95% confidence interval [CI] = 9.19 [5.67, 14.88]); the *MAPT* haplotype (*p* = 3.2 × 10^−12^; OR [95% CI] = 0.79 [0.73, 0.84]); and the *MMRN1/SNCA* locus (*p* = 4.0 × 10^−12^; OR [95% CI] = 1.41 [1.28, 1.55]) (Figure 2A).^10^ We also detected five established pathogenic *GBA1* variants that fell short of the study-wide significance threshold, including the p.Glu365Lys association (1–155236376-C-T, *p* = 4.47 × 10^−8^, OR [95% CI] = 1.61 [1.37, 1.89]) and others (Table S3).^11^

### Gene-level collapsing analysis

We have previously introduced a gene-level collapsing analysis framework that tests rare variants in aggregate, which demonstrably bolsters power for rare-variant-driven associations.^8,12,13^ In this method, we identify rare variants that meet a predefined set of criteria (that is, “qualifying variants” [QVs]) in each gene and test for their aggregate effect. We tested a total of 18,930 genes under nine different non-synonymous QV models to evaluate a range of possible genetic architectures (Table S4). There was no evidence of genomic inflation (Figure S2; median λ_GC_ = 1.01; range, 0.98, 1.03).

*GBA1* achieved study-wide significance in the UKB European analysis (Table 1; Figure 2B; Table S5). This association was most significant under the *flexnonsynmtr* QV model, which considers rare (minor allele frequency [MAF] < 0.1%) protein-truncating variants and missense variants that fall in missense-intolerant regions of the gene (*p* = 2.4 × 10^−9^; OR [95% CI], 3.54 [2.49, 5.04]). The fact that *GBA1* reached significance in the gene-level collapsing tests but not the ExWAS demonstrates the power gained by aggregating the contributions of multiple distinct rare variants within a gene. We performed a “leave-one-out” to identify which individual variants contributed most to the association under the *flexnonsynmtr* model. This analysis identified the variants 1–155236366-C-T (p.Arg368His), 1–155238260-G-C (p.Ser212*), and 1–155240629-C-T (c.115+1G>A) as most influential (Figure S3; Table S6). Notably, only two of the five *GBA1* ExWAS associations (p < 1 × 10^−4^) qualified as QVs in the *flexnonsynmtr* model. The remaining three variants, which included two previously implicated in PD (1–155236376-C-T [p.Glu365Lys] and 1–155236246-G-A [p.Thr408Met]), exceeded the model’s MAF threshold.

*ITSN1* emerged as the second highest-ranked gene in the collapsing analysis, with a suggestive (*p* = 6.1 × 10^−7^) association under the protein-truncating variant (*ptv*) model (Table 1; Figure 2B; Table S5). *ITSN1* PTVs conferred a striking effect size: carriers had 10 times greater odds of developing PD than non-carriers (OR [95% CI] = 10.53 [5.2, 21.34]). A sensitivity analysis using Firth’s logistic regression supported the robustness of this association against confounding due to sex, age, ancestry (principal components 1–4), and exome-wide PTV burden (*p* = 4.63 × 10^−7^, OR [95% CI] = 10.52 [4.82, 20.75]).

*ITSN1* PTVs were rare, with a collective case carrier frequency of 0.24% (*n* = 9 out of 3,702) compared to a control carrier frequency of 0.02% (*n* = 54 out of 233,378). The effect size for the *ITSN1* association exceeds those of previously established loci (Figure 2C), including the deleterious *LRRK2* p.Gly2019Ser variant. We re-ran the collapsing analysis for *ITSN1* after excluding individuals with potentially pathogenic variants in *GBA1* and *LRRK2*, resulting in 3,301 cases and 217,556 controls (see STAR Methods). Among the nine cases carrying a qualifying ITSN1 PTV, none had a potentially pathogenic variant in *GBA1* or *LRRK2*, whereas five of the 54 controls with ITSN1 PTVs did. As anticipated, this exclusion increased the effect size of the ITSN1 association: *p* = 2.0 × 10^−7^ (OR [95% CI] = 12.14 [5.2, 25.0]).

Several other top-ranking hits in the European collapsing analysis that did not meet our stringent *p*-value cutoff have prior functional or genetic support. These include *ADH5*, which encodes alcohol dehydrogenase, and *MERTK*, which has been implicated in the mediation of α-synuclein fibril uptake by human microglia (Table 1; Table S5).^14^ However, additional analyses are required to firmly implicate these genes as PD risk genes.

Finally, we performed a pan-ancestry collapsing analysis (Table S7). No additional genes reached significance, but future studies incorporating more cases from diverse ancestries will be crucial for further uncovering the genetic architecture of PD and ensuring equitable advances in both genetic diagnoses and targeted therapies.

### *In silico* investigation of *ITSN1* variation

Qualifying *ITSN1* PTVs were observed in 0.24% of cases compared to 0.023% of controls. There were seven unique qualifying PTVs among UKB European cases; five were singletons and two were observed in two cases each (21–33782101-C-T [p.Gln598*] and 21–33811223-G-T [c.2567+1G>T]) (Figure 2D; Table S8). These variants were scattered throughout the gene body, suggestive of a loss-of-function mechanism (Figure 2D). There are 16 impacted protein-coding *ITSN1* transcripts in Ensembl (v92). We ran the *ptv* model on each transcript individually and found no evidence of preferential signal enrichment in any single transcript. Crucially, the signal persisted in transcripts highly expressed in the nervous system^15,16^ (Table S9; Figure S4).

Consistent with the UKB control carrier frequency, the cumulative PTV allele frequency is 0.021% among non-Finnish European non-neurological controls in gnomAD v2.1.1 (*n* = 44,779 exomes) (Tables S10 and S11). We next queried our prior phenome-wide association study (PheWAS; azphewas.com)^8^ to determine if PTVs in this gene were associated with any other phenotype in the UKB. Among ~18,000 tested phenotypes, none were associated with *ITSN1* at *p* < 1 × 10^−4^. It is notable, however, that haploinsufficiency of *ITSN1* has recently been associated with autism spectrum disorder (ASD) and episodic memory decline.^17,18^

### Replication analysis

We next performed a replication analysis for the top-ranking (*p* < 1 × 10^−4^) gene-level associations in an independent dataset. Our primary replication dataset included 3,146 cases from AMP-PD and 50,754 non-neurological controls in AoU (Tables S1 and S2). The most significant collapsing analysis signal emerged from *GBA1* (*p* = 7.4 × 10^−9^; OR [95% CI] = 3.8 [2.5, 5.6]; *flexnonsynmtr* model). *ITSN1* PTVs were also significantly associated with increased PD risk (*p* = 5.4 × 10^−3^; OR [95% CI] = 7.18 [1.61, 25.75]). QVs were present in four out of 3,146 (0.13%) AMP-PD cases and nine out of 50,754 (0.02%) AoU controls (Tables S8 and S12). The signals for *GBA1* and *ITSN1* were flat (*p* > 0.05) for the negative control synonymous model, suggesting the absence of systematic biases. No other gene-level association replicated with *p* < 0.05 in this cohort (Note S1).

Carriers of *GBA1* and *LRRK2* variants were selectively recruited to the AMP-PD cohort.^19^ To determine if the overrepresentation of these individuals downwardly biased the association between PD and *ITSN1*, we reperformed the association test excluding carriers with likely pathogenic *GBA1* or *LRRK2* variants (*n* = 701 excluded cases, *n* = 1,560 excluded controls). No ITSN1 *PTV* carriers harbored a *GBA1* or *LRRK2* variant, and the odds ratio moderately increased (*p* = 2.5 × 10^−3^ and OR [95% CI] = 9.0 [2.0, 32.1]).

We sought to replicate the *ITSN1* signal further. The effect size between *ITSN1* PTVs and PD was in the same direction in the 100kGP dataset (Figure 2E), although this much smaller cohort was underpowered to detect a significant effect in isolation (Figure S5). A recent report also found an enrichment of rare (MAF < 0.1%) *ITSN1* PTVs among PD cases (*n* = 5 out of 4,668) versus controls (*n* = 36 out of 355,276) in the deCODE Icelandic population^7^ (Fisher’s exact test [FET] *p* = 1.9 × 10^−4^; OR [95% CI] = 10.6 [3.2, 27.1]). Combining evidence from the UKB and three replication cohorts, the association between *ITSN1* and PD was statistically unequivocal (Cochran-Mantel-Haenszel [CMH] test *p* = 4.54 × 10^−12^; OR [95% CI] = 9.6 [5.4, 16.0]). These analyses firmly link *ITSN1* haploinsufficiency with a markedly increased risk of PD.

### Characterizing *ITSN1*-associated PD

Strongly genetic forms of disease often present with earlier age of onset and resistance to treatment.^1,3^ We compared the age at diagnosis for *LRRK2*, *GBA1*, and *ITSN1* variant carriers to the other PD cases in the UKB EUR cohort. PD cases who did not carry a variant in one of these three genes had a median age of diagnosis of 70 (IQR, 63, 75). In contrast, the median age of diagnosis was 66 years (IQR, 60, 72) in the *GBA1 flexnonsynmtr* QV carriers (*n* = 33, *p* = 0.01) and 66 (IQR, 61, 71) in p.Gly2019Ser *LRRK2* variant carriers (*n* = 19; *p* = 0.06) (Figure S6A). The age of diagnosis also trended earlier for the nine *ITSN1* PTV carriers (median, 64; IQR, 59, 75; *p* = 0.05; Figure S6B).

Skuladottir et al. reported that PD onset was earlier for *ITSN1* PTV carriers (mean, 57.8; standard deviation, 18.0) versus noncarriers (mean, 73.0; standard deviation, 10.3) in the deCODE cohort.^7^ In AMP-PD, the median age of diagnosis was 67 (*n* = 4; IQR, 65, 69) for *ITSN1* PTV carriers versus a median of 61 (IQR, 54, 68) for the remaining cases. Compared to the UKB and deCODE, which are broadly representative of the general population, the AMP-PD and 100kGP cohorts include subsets of individuals who were recruited due to suspected genetic diseases and/or a positive family history. Altogether, these data suggest that PTVs in *ITSN1* may predispose individuals to an earlier disease onset.

Although PD tends to be more prevalent among males, this trend does not always extend to genetic forms of the disease.^20–22^ We thus assessed the rates of PD across male and female *ITSN1* PTV carriers in the UKB, AMP-PD, and 100kGP cohorts, including individuals who were originally excluded in the collapsing analysis during the sex balancing step. In total, eight out of 54 (14.8%) male carriers and six out of 42 (14.3%) female carriers were diagnosed with PD. We then used a Cox regression with Firth’s penalized likelihood to understand whether sex would predict differences in disease risk among the 96 *ITSN1* PTV carriers when modeled as an age-dependent event. There was negligible evidence for association to age of PD diagnosis in a model with sex as the only predictor (*p* = 1.00; hazard ratio [95% CI] = 1.0 [0.4, 2.9]) or when including cohort as a covariate (*p* = 0.7; hazard ratio [95% CI] = 0.8 [0.3, 2.4]) (Figure S9). Thus, PD susceptibility among *ITSN1* PTV carriers appears to be independent of sex.

Family history of neurodegenerative disease is frequent in *ITSN1* PTV carriers with PD, suggesting that some of these variants may be inherited rather than *de novo*. Among UKB Europeans, five out of nine carriers had a family history of either PD or Alzheimer’s disease/dementia in their parents or siblings. Notably, three of these individuals had a family history specifically of PD. In AMP-PD, none of the *ITSN1* cases had a recorded PD family history.

### *ITSN1* case series

We next performed an additional lookup in a local databank at Baylor College of Medicine to gain a better understanding of the presentation of PD in *ITSN1* carriers. Out of 890 multi-ancestry PD patients in this cohort, we identified three heterozygous *ITSN1* PTV carriers (c.455_456dup:p.Ala153Glnfs*43, c.1909C>T:p.Arg637*, and c.1713del:p.Leu572*). The three patients had relatively young-onset PD, with diagnosis at ages 49 (female), 50 (male), and 52 (male) years of age. One of the subjects had a recorded family history of PD (paternal aunt). All three were evaluated by movement disorders specialists and had typical PD manifestations and expected progression. Two of the patients had a tremor-dominant phenotype, and the other was noted to have the postural instability-gait difficulty PD subtype. All three were responsive to levodopa with improvement in motor manifestations, and one individual was subsequently treated with deep brain stimulation. In terms of non-motor manifestations, one patient developed anxiety and mild cognitive impairment 16 years into her disease course.

### Supporting evidence for *ITSN1* as a PD risk gene

**Figure 3.**
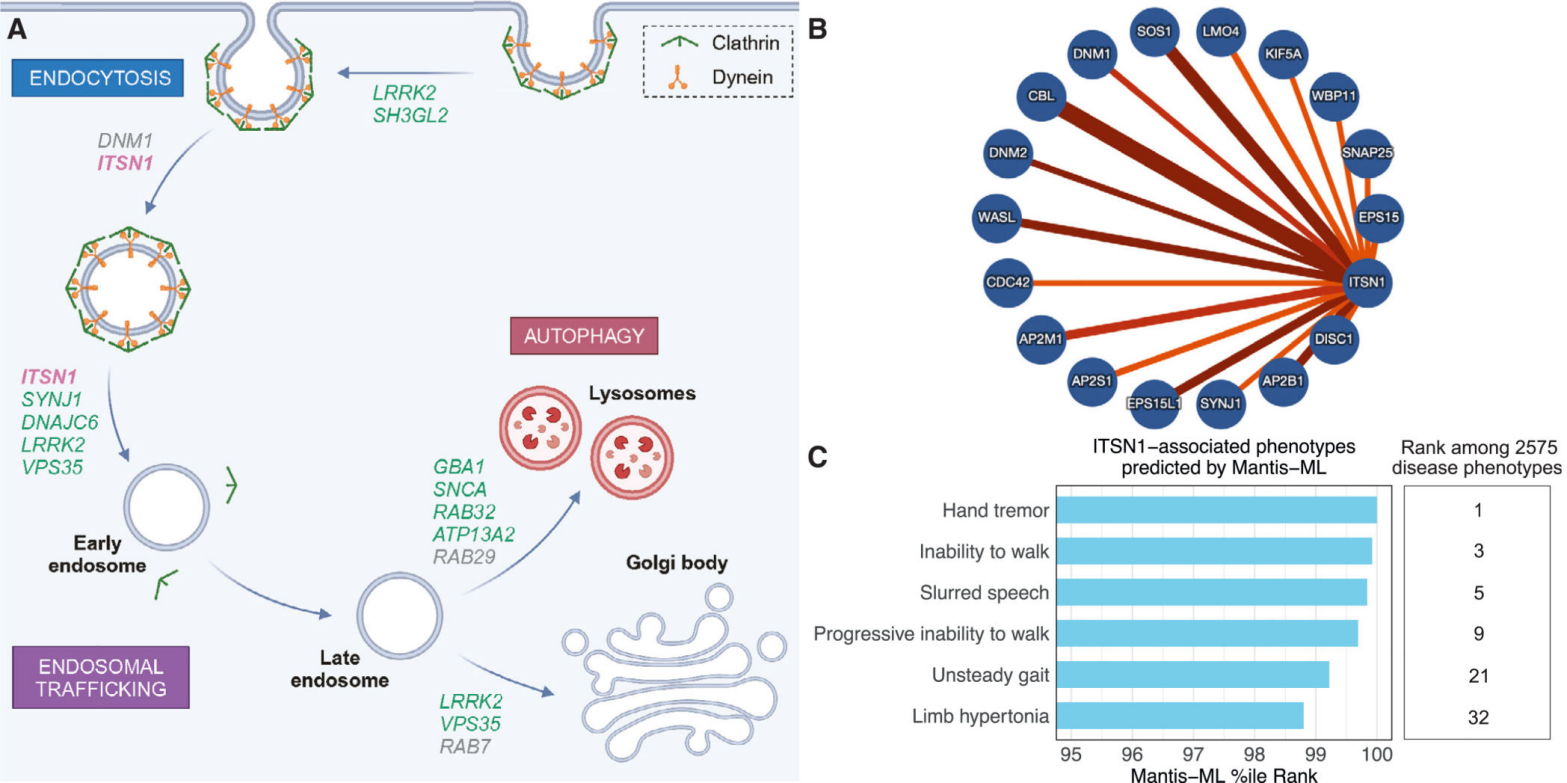
Interrogation of *ITSN1*’s function and connection to PD (A) Schematic of vesicular trafficking pathway. Previously established PD-associated genes are listed in green. (B) PPI map of ITSN1. Line width depicts confidence of the interaction. (C) Disease phenotypes ranked highly against ITSN1 by the machine-learning algorithm Mantis-ML. All shown are well-established clinical signs of parkinsonism and were ranked by Mantis-ML in the top 1.25% of predicted associations. See also Table S13.

We next analyzed protein-protein interaction (PPI) networks in IntAct,^23^ which revealed that ITSN1 physically interacts with several critical endocytic proteins (Figures 3A and 3B). This is consistent with ITSN1’s established role as a multivalent scaffold protein that organizes endocytic protein interaction networks to promote clathrin-mediated endocytosis and synaptic vesicle recycling.^24^ Interestingly, ITSN1 appears to interact with Synaptojanin-1 (*SYNJ1*), which is associated with early-onset PD. Another interactor, WASL (*WASL*), has also recently been suggested as a candidate PD gene.^25^ Overall, ITSN1 appears to be embedded in a dense, interconnected network of proteins that cooperate at virtually every stage of vesicular transport (Figure 3A).

We next looked to Mantis-ML for orthogonal support of the association between *ITSN1* and PD (https://pages.scp.astrazeneca.net/cgr/mantisml).^26^ Briefly, this semi-supervised machine-learning framework estimates the probability of genes being associated with different phenotypes included in the Human Phenotype Ontology, using known disease-associated genes as “seed genes.” It leverages numerous features, including a knowledge graph, gene expression data, reported mouse phenotypes, and genic intolerance. Across 2,575 studied phenotypes, hand tremor, inability to walk, and slurred speech were among the top 10 Mantis-ML predictions (Figure 3C; Table S13). The scores for the top six phenotypes PD-related phenotypes were >2 standard deviations greater than the average score for the remaining phenotype predictions for *ITSN1*. Since rankings from Mantis-ML are distinct from our genetic association analyses, these findings provide additional support for the association between *ITSN1* and PD.

### Functional investigation of *ITSN1*

**Figure 4.**
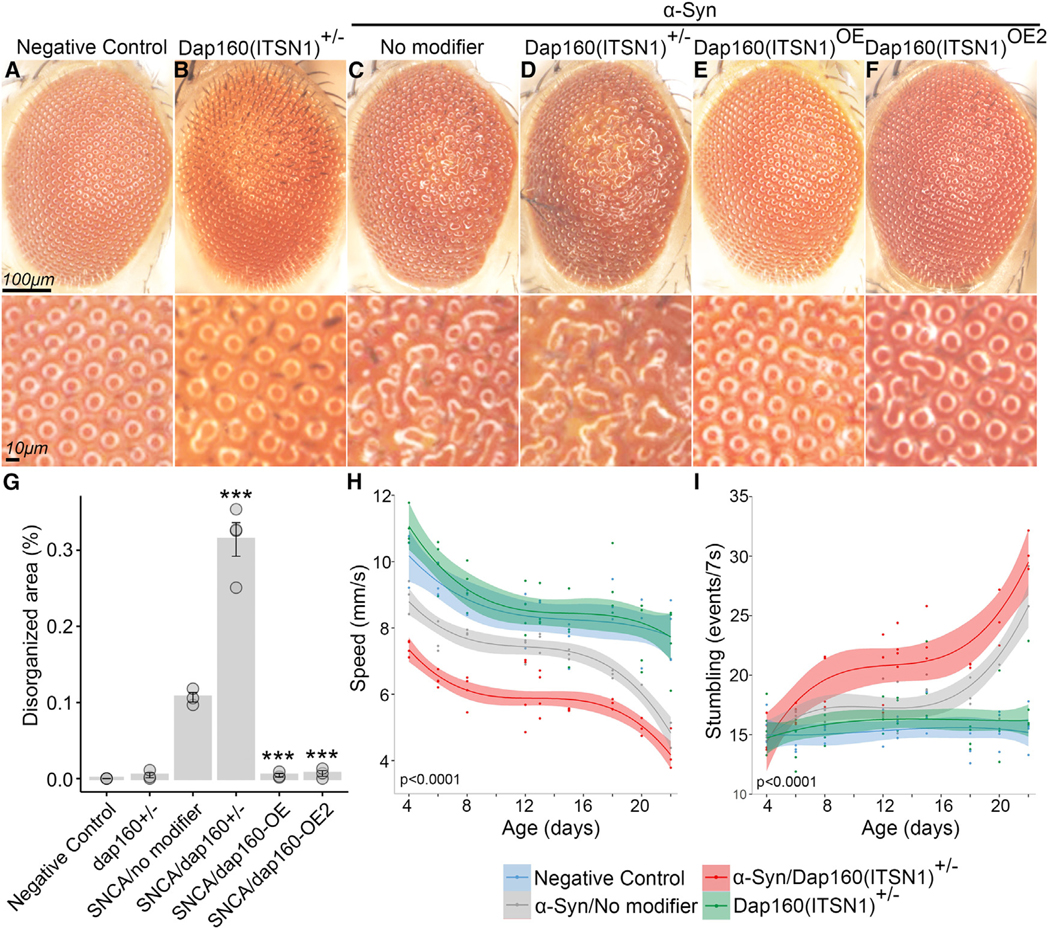
*Dap160(ITSN1)* modulates α-synuclein-induced neurodegeneration in *Drosophila* (A–F) Representative images of *Drosophila* eyes among the following genotypes: wild type (A), *Dap160*^*+/*−^ (B), α-Syn/no modifier (C), α-Syn/Dap160^+/−^ (D), and two independent *Dap160* overexpression alleles in the α-Syn background. Scale bar, 100 μm in the top row and 10μm in the bottom row. (G) Quantification of the disorganized eye area as a percentage of total eye area for the genotypes shown in (A)–(F). Bar represents the mean across three flies per genotype; error bars represent standard error of the mean. *p* values were generated via ANOVA followed by Dunnett’s test using α-Syn/no modifier as control (***p < 0.001). (H and I) Motor performance as a function of age measured via speed (H) and number of stumbling events (I). Each point represents the average speed for a given replicate (*n* = 10 flies per replicate; see STAR Methods). Lines represent spline fits, and shaded areas represent 95% confidence intervals. *p* values were obtained using a nonlinear random mixed-effect model ANOVA applied to spline regressions of α-Syn/no modifier vs. α-Syn/Dap160^+/−^. α-Syn, α-synuclein; OE, overexpression. See Table S14 for full genotype information.

We and others have used transgenic *Drosophila* lines expressing human α-synuclein to study how genes associated with PD interact with α-synuclein-mediated neurotoxic mechanisms. Here, we used this model^27,28^ to functionally characterize the effects of *ITSN1* haploinsufficiency and investigate its potential genetic interaction with α-synuclein. *Drosophila* expressing α-synuclein exhibit photoreceptor neuron degeneration, which appears as disorganization of the external hexagonal facets (Figure 4A). This “rough-eye” phenotype has been observed in *Drosophila* models of neurodegeneration, including Alzheimer’s disease and PD.^29,30^ Flies carrying a heterozygous null variant in *Dap160* (the *Drosophila* ortholog of *ITSN1*)^31^ had exacerbated α-synuclein-induced degeneration, with more severe disorganization covering a larger eye area (Figures 4C and 4D). Strikingly, overexpression of *Dap160* ameliorated the α-synuclein-induced rough-eye phenotype (Figures 4E and 4F).

We next studied the interaction of *Dap160* with α-synuclein using a locomotor assay to assess neuronal dysfunction. Consistent with prior studies, neuronal expression of α-synuclein in *Drosophila* led to a loss of climbing speed and increased stumbling events as the flies aged compared with age-matched controls (Figures 4F and 4G). Heterozygous loss of *Dap160* worsened the phenotypes of α-synuclein-expressing flies across all ages (Figures 4F and 4G). Interestingly, *Dap160*^*+/*−^ flies not expressing α-synuclein displayed no eye or motor impairments, suggesting a synergistic interaction between α-synuclein and *Dap160* haploinsufficiency.

**Figure 5.**
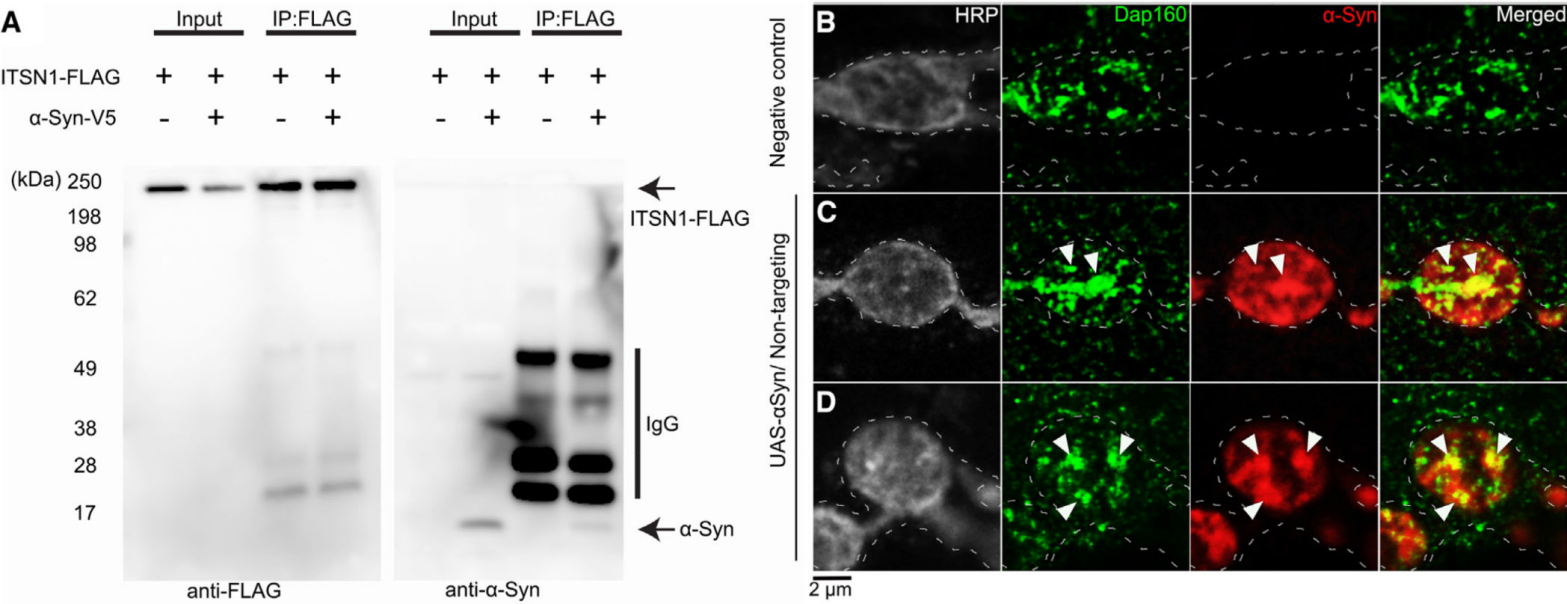
Molecular interaction between ITSN1 and α-synuclein (A) Immunoblots of lysates (input) and FLAG immunoprecipitates (IPs) from HEK293T cells overexpressing FLAG-tagged ITSN1 and α-Syn. Data are representative of *n* = 3 independent experiments. (B–D) Immunofluorescence against α-Syn (red) and Dap160 (green) in the synaptic boutons of *Drosophila* neuromuscular junctions from wild-type larvae (B) or larvae expressing α-Syn (C and D). Arrowheads indicate colocalization events. Scale bar, 2 μm. Data are representative of *n* = 245 neuromuscular junctions across 18 α-Syn and 14 wild-type larvae.

We next assessed whether α-synuclein and ITSN1 interact at the molecular level. First, we performed a coimmunoprecipitation (coIP) assay in HEK293T cells co-expressing FLAG-tagged ITSN1 and V5-tagged α-synuclein. Western blot analysis showed thatα-synuclein-V5 co-precipitated with ITSN1-FLAG (Figure 5A). We further investigated the ITSN1-α-synuclein interaction by immunofluorescence (IF) in *Drosophila*. Because Dap160 localizes to synaptic boutons,^31,32^ we co-stained both proteins in *Drosophila* larval neuromuscular junctions. Dap160 co-localized with α-synuclein aggregates in 42.9% of 245 analyzed synaptic boutons (Figures 5B–5D). Altogether, these results support an interaction between ITSN1 and α-synuclein, further supporting the role of ITSN1 haploinsufficiency in mediating PD risk.

## DISCUSSION

This study leveraged multiple population-level datasets to assess the genetic architecture of PD, with a particular emphasis on rare variation. Our variant- and gene-based association tests not only recapitulated multiple known associations but also uncovered an association driven by ultra-rare PTVs in *ITSN1*. This association was unequivocally significant in a combined analysis across all four cohorts (Figure 2). In the UKB, *ITSN1* PTV carriers were at an over 10-fold increased risk of PD, ranking higher than every other detected risk locus. Our functional studies in *Drosophila* demonstrated that haploinsufficiency of the *ITSN1* homolog *Dap160* exacerbates α-synuclein-induced compound eye degeneration and locomotor dysfunction (Figure 4). Prior evidence has also shown that *Itsn1* null mice exhibit PD-related phenotypes, including abnormal gait, limb grasping deficits, and decreased prepulse inhibition.^33^ Collectively, these data establish *ITSN1* as a genetic cause of PD and suggest that it may interact with α-synuclein-mediated pathogenic mechanisms.

There has been ongoing research into rare-variant-driven associations with PD.^6,7,34,35^ Two recent studies^34,35^ focused on familial PD have independently converged on a rare variant in *RAB32* that was not sufficiently common in UKB to be included in our ExWAS. One of these studies^35^ included an exome-wide gene-level burden analysis but did not detect an association with *ITSN1*. One possible explanation for this is that the authors only ran a model that combined PTVs and missense variants, which may have obscured the PTV-specific effect. Another recent study performed a large, multi-cohort meta-analysis on rare protein-coding variation in 7,184 cases, 6,701 family-history-based proxy cases, and 51,650 controls.^6^ While this study reported several candidate PD genes, *ITSN1* was not among them. This could, in part, be attributed to the more homogeneous and 4.5 times larger control cohort in our discovery analysis. The diversity of findings across these studies underscores the importance of employing a range of approaches and leveraging large sample sizes to fully elucidate the complex genetic architecture of PD.

*ITSN1* encodes a multidomain scaffold protein that orchestrates protein-protein interactions during clathrin-mediated endocytosis and synaptic vesicle recycling.^36^ Notably, several other PD risk genes function in synaptic vesicle trafficking, including *SNCA*, *LRRK2*, *DNAJC6*, *SYNJ1*, *VPS35*, and *SH3GL2*.^37^ Dysfunction of this pathway has been implicated in multiple aspects of PD pathogenesis, such as the reduced degradation, increased aggregation, and missorting of α-synuclein.^38^ Additionally, defects in synaptic vesicle endocytosis can disrupt the precise temporal coupling of exocytosis and endocytosis required for sustained neurotransmission, potentially contributing to synaptic dysfunction and neuronal loss in PD.^39^ Given its role as a scaffold protein, *ITSN1* haploinsufficiency may exacerbate these processes by destabilizing endocytic protein networks and impairing vesicle recycling. This is shown by decreased endocytosis rate and abnormal endosomes in *Itsn1* null mice,^40^ as well as by reduced localization of critical endocytic proteins, enlarged vesicles, and aberrant synaptic formation in *Dap160* null *Drosophila*.^31^ However, further studies are needed to elucidate the precise role of *ITSN1* in human PD pathogenesis and its potential as a therapeutic target.

*ITSN1* haploinsufficiency has also recently been implicated in moderate forms of ASD.^17^ Other studies have highlighted a convergence between ASD and PD, with epidemiologic data suggesting that people with ASD are three times more likely to develop parkinsonism.^41^ Synaptic transmission dysfunction is emerging as a convergent mechanism between neurodevelopmental and neurodegenerative disease.^42,43^ Interestingly, the association between *ITSN1* and ASD was driven in part by rare PTVs that probands inherited from unaffected parents. The spectrum of phenotypes associated with *ITSN1* haploinsufficiency, ranging from apparently unaffected carriers to individuals with increased risk of childhood-onset ASD or adult-onset neurodegenerative diseases, suggests variable penetrance and/or expressivity of *ITSN1* mutations. *ITSN1* has also recently been associated with episodic memory decline/Alzheimer’s disease.^18^ In *Drosophila*, we have observed that *Dap160* loss exacerbates locomotor phenotypes in Tau and secreted Aβ42 expressing models as well (data not shown). We propose that rare variants in *ITSN1* might lead to different phenotypic outcomes depending on additional genetic background, environmental exposures, or epigenetic modifications. Future studies aimed at identifying these modifiers would be critical for prognostication and could also unveil potential therapeutic avenues for mitigating *ITSN1*-mediated clinical disease.

Overall, our findings add to the growing body of evidence implicating synaptic vesicle trafficking defects in PD and highlight ITSN1 as a potential therapeutic target. Importantly, this study also underscores the immense value of large-scale sequencing efforts for identifying rare-variant contributions to complex diseases like PD.

### Limitations of the study

Small sample sizes limited our ability to detect associations among individual non-European populations, and there is clear evidence that population-specific variation can exert substantial risk for PD.^44^ It is critical that we continue expanding sequencing efforts to improve the representation of non-European ancestries, both for health equity and for fully elucidating the genetic architecture of human disease. Additionally, our data from *Drosophila* and HEK293T cells provide evidence of an interaction between ITSN1 and α-synuclein, offering important initial insights into disease mechanism. However, it will be crucial to validate and build upon these preliminary findings in more physiologically relevant model systems.

## RESOURCE AVAILABILITY

### Lead contact

Further information and requests for resources and reagents should be directed to and will be fulfilled by the lead contact: Ryan Dhindsa (ryan. dhindsa@bcm.edu).

### Materials availability

This study did not generate new unique reagents.

### Data and code availability

- Primary genetic association tests were performed using PEACOK (version 1.0.7). PEACOK 1.0.7 is available on Zenodo^45^ (https://doi.org/10.5281/zenodo.7097303) and GitHub (https://github.com/astrazeneca-cgr-publications/PEACOK/).
- Variant- and gene-level summary statistics are available in the supplemental tables and on Zenodo^46^ (https://doi.org/10.5281/zenodo.14705134).
- Microscopy data reported in this paper will be shared by the lead contact upon request.
- Any additional information required to reanalyze the data reported in this paper is available from the lead contact upon request.

## STAR★METHODS

### EXPERIMENTAL MODEL AND STUDY PARTICIPANT DETAILS

#### Human study participants

The discovery cohort comprised of 490,560 multi-ancestry UK Biobank (UKB) participants. Using available ICD-10 codes and self-reported data, we identified 3,809 PD cases and 247,101 controls without any reported history of neurological disorders (Table S1). Among the UKB cohort, 3,702 cases and 233,378 controls were of European ancestry. We also analyzed three replication cohorts comprised of individuals of European ancestry. One included 3,739 cases from the Accelerating Medicines Partnership program for Parkinson’s disease (AMP-PD) and 50,754 non-neurological controls from All of Us (AoU). The Accelerating Medicine Partnership (AMP) Parkinson’s Disease (AMP PD) dataset includes whole-genome sequencing data from multiple datasets, including the Parkinson’s Progression Markers Initiative (PPMI), the Parkinson’s Disease Biomarkers Program (PDBP) and the Harvard Biomarker Study (HBS), BioFIND, SURE-PD3, and STEADY-PD3. The second replication dataset included 593 cases and 7,402 controls from the 100,000 Genomes Project (100kGP).^54^ The third replication cohort consisted of 4,668 cases and 355,276 controls from a recently published rare variant PD study in the Icelandic deCODE genetics cohort.^7^ As with the UKB, controls in the replication datasets were restricted to individuals with no history of neurological disease in their health records (assessed via ICD-9/ICD-10 codes). To reduce confounding by sex, we downsampled the controls to achieve the same male:female ratio observed in cases. These cohorts have all been previously described, and full demographic details of all cohorts are included in Table S2. The appropriate institutional review boards of the participating institutions approved each study, and informed consent was obtained from all subjects or their surrogate decision-makers.

To identify additional PD cases with *ITSN1* PTVs, we accessed whole-exome sequencing data from two different cohorts at Baylor College of Medicine (BCM), totaling 890 unique PD cases. One cohort has been described previously.^56^ The other cohort included 816 subjects who were recruited from the Parkinson’s Disease Center and Movement Disorders Clinic and Alzheimer’s Disease and Memory Disorders Clinic at Baylor College of Medicine, and from National Alzheimer Center (Nantz) at the Houston Methodist Hospital, during routine clinic visits between 2022 and 2024. PD and Alzheimer’s disease (AD) patients were diagnosed by a board certified neurologic clinical specialist in movement disorders and dementia. Among the newly sequenced cases, 530 were male and 286 were female. The median age at enrollment was 65 and a majority of participants (*n* = 672; 82%) were of European ancestry. All participants agreed to be part of the Biorepository study, which is a collection of clinical information and blood or saliva samples of both patients with neurodegenerative disorders and controls. Demographics and selected clinical details were collected through chart review and confirmed based on an unstructured interview at the time of sample collection in the clinic, including family history of PD (defined as any affected blood relative), age at symptom onset, and results from cognitive screening. All subjects provided informed consent. The institutional review boards at Baylor College of Medicine and the Houston Methodist Hospital approved both the biospecimen collection and sequencing.

#### Drosophila

All strains were obtained from the Bloomington Drosophila Stock Center (https://bdsc.indiana.edu/Home/Search) and included in the key resources table. To express human wild-type codon optimized α-synuclein, we used BDSC UAS line 51375 (P{w[+mC] = UAS-SNCA.J}1) in combination with the pan-neuronal post mitotic driver elav-GAL4(C155) (P{w[+mW.hs] = GawB}elav[C155], BDSC-458) or the eye driver GMR-GAL4 (P{GAL4-ninaE.GMR}12, BDSC-1104). To modulate expression of *Dap160*, the Drosophila homolog of *ITSN1*, we used the amorphic mutant lines Dap160Δ1 (BDSC-24877) and Dap160Δ2 (P{w[+mC] = UAS-Dap160.K}4.1, BDSC-24878), and the UAS-Dap160 overexpression line BSDC-42695. Genotypes are also included in Table S13. Age-dependent motor performance was assessed using 10 age-matched females per-replica per-genotype as previously described.^57,58^ Females were collected over a 24-h period and transferred into new media (20g yeast, 20g Tryptone, 30g sucrose, 60g Glucose, 0.5g MgSO_4_.7H_2_O, 0.5g CaCl_2_.2H_2_O, 80g Inactive Yeast, 1L H_2_O) daily. Animals were kept at 25°C. Motor performance assays were performed from days 4–22. For eye imaging, the animals were raised at 29°C. Eye images were captured from adult (1-day old) female flies (*n* = 3 flies per genotype). Neuromuscular junction images were taken at the larval stage. Larvae were raised at 28°C. We imaged 245 NMJs across a total of 18 Synuclein-expressing larvae and 14 wild type flies.

#### Cell lines

HEK293T (293T) cells (obtained and certified from ATCC) were cultured in DMEM (Thermo Fisher Scientific, MT10013CV) containing 10% FBS and Antibiotic-Antimycotic (Gibco, 15240096) at 37°C and 5% CO_2_. HEK cells were not tested for mycoplasma.

### METHOD DETAILS

#### Sample-level quality control

To perform genetic association tests, we defined cohorts of people with PD (cases) and people without any neurological disorder (controls) from four datasets (Table S1).^19,48,50,53,54^ Samples retained for analysis were determined by per-dataset quality control (QC) protocols described below.

##### UK biobank

Whole-genome sequencing (WGS) data of the UKB participants were generated by deCODE Genetics and the Wellcome Trust Sanger Institute as part of a public-private partnership involving AstraZeneca, Amgen, GlaxoSmithKline, Johnson & Johnson, Wellcome Trust Sanger, UK Research and Innovation, and the UKB. The WGS sequencing methods have been previously described.^48,59^ Briefly, genomic DNA underwent paired-end sequencing on Illumina NovaSeq6000 instruments with a read length of 2 × 151 and an average coverage of 32.5x. Conversion of sequencing data in BCL format to FASTQ format and the assignments of paired-end sequence reads to samples were based on 10-base barcodes, using bcl2fastq v2.19.0. Initial quality control was performed by deCODE and Wellcome Sanger, which included sex discordance, contamination, unresolved duplicate sequences, and discordance with microarray genotyping data checks.

UK Biobank genomes were processed at AstraZeneca using the provided CRAM format files. A custom-built Amazon Web Services cloud compute platform running Illumina DRAGEN Bio-IT Platform Germline Pipeline v3.7.8 was used to align the reads to the GRCh38 genome reference and to call small variants. Variants were annotated against the transcript for which they are most damaging using SnpEff v4.3^60^ and Ensembl Build 38.92.^61^ Phenotypic data used to identify people with Parkinson’s disease (cases) and non-neurological controls (see Table S1) were accessed in April 2022.

Sample-level filters were applied as previously described to derive a subset of UKB suitable for analysis.^8^ Briefly, we removed any person withdrawn from UKB and without linked WGS data. Where available, WGS data were checked for concordance with previous exome sequencing and genotyping array data releases using the KING relatedness software v2.2.3,^62^ excluding people with <0.49 estimated kinship with their exome-sequencing or <0.465 with the array data. Of the remaining samples, we retained only those with VerifyBamID FREEMIX^52^ contamination <0.04 and ≥10x coverage across ≥94.5% of Consensus Coding Sequence (CCDS) database (release 22) bases.^49^ The cohort was filtered further using the *ukb_gen_samples_to_remove* function from the ukbtools (v0.11.3)^63^ R package to select the largest subset of individuals with ≤0.3536 kinship estimate from KING, removing any first-degree relatives.

The data were next stratified by inferred ancestry across four superpopulation cohorts: African (AFR), East Asian (EAS), European (EUR), and South Asian (SAS). Ancestry was inferred based on the 1000 Genomes phase 3 cohort^64^ using the Peddy (v0.4.2) software package^65^ and we retained people with ≥0.90 probability of belonging to an ancestry group. For EUR ancestry, which represents >90% of the dataset, we further restricted to people ≤4 standard deviations (SD) from the mean of the first four genetic principal components (PCs). To avoid analytical confounding, female controls were pseudo-randomly downsampled if there was a significant difference in the odds of being male across cases and controls from a given ancestry cohort (Fisher’s Exact Test *p*-value <0.05). Table S1 overviews per-ancestry cohorts before and after sex rebalancing, where applied.

##### AMP-PD

WGS data for cohorts contributing to AMP-PD were generated by Macrogen and the Uniformed Services University of Health Sciences using the Illumina HiSeq X Ten sequencer with samples coming from whole blood. Data processing was performed on the Google Cloud Platform and in Terra (https://app.terra.bio/) against Build 38 of the Human Genome reference (GRCh38DH, 1000 Genomes Project version). We accessed the joint-genotyping v4 release in April 2024. The joint genotyping protocol has been previously described.^19^ Varaints were annotated against multiple transcripts with the Ensembl Variant Effect Predictor (VEP)^66^ and converting to Variant Transforms format.

AMP-PD WGS samples passing the following QC protocol, which has also been described previously,^19^ were used in joint-genotyping. Samples were retained based on the following inclusion criteria: VerifyBamID FREEMIX ≤0.03, Mean coverage ≥25 reads per variant, joint calling missingness <0.05, Transition transversion ratio (TiTv) ≥2 for variants in dbSNP (v138), deviation from expected autosomal homozygous genotype counts (Absolute method-of-moments F ≤ 0.15). Sex was inferred from genetic data, and samples with an explicit mismatch to self-reported sex (‘male’ or ‘female’) were removed.

The KING relatedness software was used for estimation of relatedness in the cohort and to infer ancestry. Concordance between WGS and NeuroX genotyping array^51^ samples was tested where available, excluding people with matched samples predicted to be less closely related than duplicates/monozygotic twins. A deduplication step was performed across the WGS samples, excluding the lower-quality WGS sample from each pair predicted to be duplicates/monozygotic twins.

Ancestry was inferred based on principal component analysis against the HapMap 3 reference panel.^67^ Continental ancestries were assigned to people within ±6 standard deviations of the mean of PC1 and PC2 for that population in the HapMap reference. Individuals with discrepant clinically reported race and genetically inferred ancestry were removed.

##### All of Us

At the time that this study was conducted, All of Us contained short-read WGS data from 245,376 individuals, 123,063 of whom were classified as having European genetic ancestry. Full details on genome sequencing and downstream quality control have been previously published.^53^ Briefly, PCR-free Barcoded WGS libraries on the Illumina NovaSeq 6000. Following demultiplexing, initial QC analysis was conducted using the Illumina DRAGEN pipeline. All of Us samples passing the following quality control metrics were used in joint calling and released to the research community: mean coverage ≥30x, genome coverage ≥90% at 20x, All of Us Hereditary Disease Risk gene coverage ≥95% at 20x, aligned Q30 bases ≥8 × 10^10^, and contamination ≤1%. Additional QC checks included fingerprint mismatch and array mismatch. We annotated using SnpEff v4.3.

Ancestry was inferred across populations defined in gnomAD using a random forest classifier trained using a diverse reference dataset of 3,202 samples and 151,159 autosomal single-nucleotide polymorphisms. By projecting the All of Us samples onto the classifier’s 16-dimensional PCA space, categorical ancestry predictions were generated. Since data from AoU were used as a non-neurological control cohort for analysis together with cases of European ancestry from AMP-PD, we restricted to those classified as having European ancestry. The dataset was then restricted to include only those with corresponding sex at birth and genetically-inferred sex, resulting in 119,089 samples.

We identified pairs of related individuals using the relatedness.tsv file provided by AoU, which lists all pairs of samples with a kinship score above 0.2 (calculated via the pc_relate function in Hail). We removed the first sample of each related pair, leaving 114,415 unrelated individuals. A final dataset of 50,754 people remained after removing samples with any diagnosis under the ICD-10 neurological disease chapter (Table S1).

##### 100kGP

Whole-genome sequencing data were generated, as previously described, using TruSeq DNA polymerase-chain-reaction (PCR)–free sample preparation kit (Illumina) on the HiSeq2500 platform. Reads were aligned using the Isaac Genome Alignment Software, and the Platypus variant caller was used for small variant calling.^68^ Variants were annotated using VEP (v105) with the gnomAD plugin included.^66^ We imposed the following inclusion criteria: VerifyBamID FREEMIX contamination ≤0.03; aligned reads have ≥15x coverage across 95% of the genome with MQ > 10; >90% concordance between variant calls from sequencing and matched genotyping array; median fragment size >250bp; <5% chimeric reads; >60% mapped reads; <10% AT dropout; concordance between self-reported and genetically determined sex.

Ancestry was inferred by training a random forest model upon PCs 1–8 from the 1000 Genomes phase 3 cohort.^64^ People with >99% probability of European ancestry and <4SD from the mean of PCs 1–4 in the 100kGP inferred-European cohort were retained for analysis. Finally, we removed one from each pair of individuals estimated to have R2nd degree relatedness (kinship coefficient >0.0442) under the plink2 implementation of the KING-relatedness software. Here we followed a 3-step procedure, removing one from each pair of related cases with the *ukb_gen_samples_to_remove* R function, removing controls related to remaining cases, and finally using *ukb_gen_samples_to_remove* to removing one from each related pair of remaining controls. Sex-rebalancing was then performed as for the UK Biobank dataset so that the odds of being male was comparable in cases and controls.

#### Variant-level association tests

We performed a variant-level exome-wide association study (ExWAS) to test for associations between PD and protein-coding variants observed in at least six participants of European ancestry in the UKB. We applied our previously described protocol to generate variant-level statistics. Variants were required to pass the following quality control criteria: coverage ≥10x; ≥0.20 of reads are for the alternate allele for heterozygous genotype calls; binomial test of alternate allele proportion departure from 50% in heterozygous state p ≥ 1 × 10^−6^; GQ ≥ 20; Fisher Strand Bias (FS) ≤200 for indels and ≤60 for SNVs; root-mean-square mapping quality (MQ) ≥40; QUAL ≥30; read position rank sum score (RPRS) ≥−2; mapping quality rank score (MQRS) ≥−8; DRAGEN variant status = PASS; ≤10% of the cohort have missing genotypes. Additional out-of-sample QC filters were also imposed based on the gnomAD v2.1.1 exomes (GRCh38 liftover) dataset.^55^ The site of all variants were required to have ≥10x coverage in ≥30% of gnomAD exomes and, if present, the variant was required to have an allele count ≥50% of the raw allele count. Variants with missing values for any filter were retained unless they failed another metric. Variants failing QC in >20,000 people, were also removed. *p* values were generated via Fisher’s exact two-sided test. Three distinct genetic models were studied for binary traits: allelic (A versus B allele), dominant (AA + AB versus BB), and recessive (AA versus AB + BB), where A denotes the alternative allele and B denotes the reference allele. Variants with minor allele frequency (MAF) ≥0.001 and p < 1 × 10^−4^ were clumped into independent loci using plink2 (v5.10 final)^47^ under default parameters (‘–clump-r2 0.5’ and ‘–clump-kb 250’), based on linkage disequilibrium in a random subsample of 10,000 individuals from UKB EUR.

#### Gene-level collapsing analysis

##### UK biobank collapsing analysis

We performed our previously described collapsing analysis framework to identify gene-level associations with PD.^8,12,69^ To perform collapsing analyses, we aggregated variants within each gene that fit a given set of criteria (i.e., “qualifying variants” or “QVs”). We employed 10 different QV models, including one synonymous model as an empirical negative control (Figure 1B; Table S2; Table S15). Nine of the collapsing models were dominant, and one was recessive. For the dominant collapsing models, the carriers of at least one QV in a gene were compared to the noncarriers. In the recessive model, individuals with two copies of QVs in either homozygous or putatively compound heterozygous form were compared to the noncarriers. Hemizygous genotypes for X chromosome genes also qualified for the recessive model.

For all models, we applied the following variant-level QC filters: coverage ≥10x; present in CCDS (release 22)^49^; ≥0.8 of reads are for the alternate allele among homozygous genotypes; ≥0.25 and ≤0.8 of reads are for the alternate allele for heterozygous genotype calls; binomial test of alternate allele proportion departure from 50% in heterozygous state p ≥ 1 × 10^−6^; GQ ≥ 20; FS ≤ 200 for indels and ≤60 for SNVs; MQ ≥ 40; QUAL ≥30; RPRS R−2; MQRS ≥−8; DRAGEN variant status = PASS. Additional out-of-sample QC filters were also imposed based on the gnomAD v2.1.1 exomes (GRCh38 liftover) dataset.^55^ For all variants, the site had to have ≥10x coverage in ≥25% of gnomAD exomes. For variants present in gnomAD, we retained those with RPRS ≥−2 and MQ ≥ 30. Variants with missing values for a particular filter were retained unless they failed another metric.

We performed these collapsing analyses across 18,930 genes in each of the four superpopulation cohorts (i.e., the European, South Asian, African, East Asian ancestry groups). *p*-values were generated via a two-tailed Fisher’s exact test. We considered the European subset for discovery analyses, as individuals of predominantly European ancestry make up more than 90% of the cohort. As a secondary analysis, we also implemented Firth’s logistic regression with the R logistf package and function (v1.26.0) to confirm that the association between PD and *ITSN1* in UKB EUR was robust to possible confounding due to age (field 21022), sex (field 35), ancestry principal components[1–4], and exome-wide PTV burden (i.e., the number of genes with at least one qualifying variant under the ptv collapsing model). Finally, to combine results across ancestries (i.e., the “pan-ancestry analysis”), we implemented a two-sided exact Cochran-Mantel-Haenszel (CMH) test with the R stats package *mantelhaen.test function* (with setting exact = TRUE). While Fisher’s exact test is well-suited for rare-variant analysis in a single cohort, CMH is designed to analyze 2 × 2 contingency tables stratified by, and controlling for, possible confounding.

##### AMP-PD + AoU collapsing analysis

We performed replication analysis for the top-ranking (p < 1 × 10^−4^) gene-level associations from the UKB in a primary replication dataset that consisted of 3,146 AMP-PD cases and 50,754 non-neurological controls in All of Us (AoU). For AMP-PD, we included variants that passed GATK QC filters and fell in CCDS regions (release 22).^49^ For the “Internal MAF” parameter (see Table S2), variants were filtered based on minor allele frequencies (MAFs) observed in the European AoU controls to ensure that filtering was not influenced by variant frequencies in people with PD. Variants absent in AoU controls but present in AMP-PD cases passed this filter automatically.

For AoU, we included variants that were met the following variant-level QC metrics: GQ ≥ 20, DP ≥ 10, AB ≥ 0.2 for heterozygotes, ExcessHet <54.69 and QUAL ≥60 for single nucleotide variants and QUAL R69 for indels. We restricted to variants in positions overlapping CCDS transcripts (release 22). We calculated *p*-values via a two-tailed Fisher’s exact test as implemented in the R stats package (v4.1.0) *fisher.test* function. We have received an exception to the Data and Statistics Dissemination Policy from the *All of Us* Resource Access Board to demonstrate the results of our collapsing analysis using All of Us short-read WGS data. To combine these results with the top-ranking UKB associations (i.e., those with p < 1 × 10^−4^), we performed an exact CMH test.

##### 100kGP collapsing analysis

For the 100kGP dataset, we focused on replicating the *ITSN1* ‘ptv’ signal. We retained variants with the following metrics: missingness %0.05; median coverage R10x; Median GQ R 15; R0.25 heterozygous allele calls do not show significant (*p* < 0.01) allele imbalance in binomial test; R0.50 complete genotype data for a variant; Hardy-Weinberg equilibrium mid p > 1 × 10^−5^ among unrelated samples of European ancestry. We tested gene-level associations to *ITSN1* under the *ptv* model (see Table S2). Variants were mapped to consequences based on their impact upon the Matched Annotation from NCBI and EMBL-EBI (MANE) transcript for the gene (ENST00000381318).

##### ITSN1 *meta-analysis*

We performed an exact CMH test to evaluate the combined evidence of association between *ITSN1* and PD across the UKB EUR, AMP-PD + AoU, 100kGP, and deCODE cohorts (*n* = 12,702 cases and 646,810 controls) under the ‘ptv’ collapsing analysis model. The deCODE stratum was derived based on summary statistics from another recent PD collapsing analysis that considered rare (MAF<0.1%) PTVs in this population.^7^

##### *Filtering of* GBA1 *and* LRRK2 *variant carriers*

We repeated the *ITSN1 ptv* model association analysis in the UK Biobank and the ‘AMP-PD + AoU’ cohorts after excluding people with potential disease-causing variants in *LRRK2* and *GBA1* to confirm the independence of the association from these genetic effects. We defined potentially disease-causing variant as those meeting any of the following criteria.

1. Reported as pathogenic or likely pathogenic in ClinVar (09 Oct 2024 release)
2. Associated at p < 1 × 10^−4^ in the ExWAS
3. Qualifying in the ‘*flexnonsynmtr*’ collapsing model for *LRRK2* or *GBA1*
4. Qualifying in the ‘*flexdmg*’ collapsing model of *LRRK2* or *GBA1*

The ClinVar and variant-level analysis criteria aim to broadly capture variants directly implicated in the disease. The collapsing-model criteria were included to capture rare variation that may cause disease but are unlikely to have been individually characterized. The *flexnonsynmtr* and *flexdmg* models specifically capture a broad range of non-synonymous variation, with a minor allele frequency threshold more lenient than other models. For the ‘AMP-PD + All of Us’ cohort, we implemented criteria 1,3, and 4 only. A small number of AMP-PD cases identified as *LRRK2* or *GBA1* variant carriers during their clinical assessment were also excluded.

#### Age of diagnosis and family history analyses

Age of PD diagnosis was ascertained for UKB cases as the interval between date of birth and of first occurrence for any code mapped to ICD-10 G20 (UKB field 131022), using the R *lubridate* package (v1.9.2). Date of birth was determined from month and year of birth (UKB fields 52 and 34), setting the day of the month be the 15^th^. Family disease histories were assessed in terms of self-reported illnesses of the father, mother, and siblings (UKB fields 20107, 20110, and 20111). Age of censoring for PD in controls was calculated as above based on the interval between date of birth and the date of most recent medical history record (which is either the date of last update for electronic health records (2021–09-30) or date of death for those who are deceased (UKB field 40000)).

For the AMP-PD cases, we estimated age of diagnosis as the earliest reported age of diagnosis for ‘Parkinson’s disease’ or ‘Idiopathic PD’ in the PD_Medical_History.csv file. Family history status was defined by an absence of any family history for PD among biological parents or ‘any other relative’ as reported in the Family_History_PD.csv file. Age of censoring for AoU controls was determined by subtracting each person’s birth year from a censor year of 2022.

For the 100kGP dataset, we determined age of diagnosis for all people with PD based on their year of birth and the earliest date that they received either an ICD-10 G20 diagnosis or were recruited for ‘Early Onset and familial Parkinson’s Disease’ proband. We derived the age of censoring for PD in two unaffected controls with an ITSN1 PTV based on their year of birth and a censor year of 2022, reflecting the end dates for Hospital Episode Statistics in 100kGP release 18.

We tested associations with age of PD diagnosis under a Time-To-Event analysis framework. Log rank tests, implemented with the R survival package (v3.7.0) *survdiff* function under default settings, were used to compare survival curves among particular genetic PD subsets against the remainder of people with PD from the UKB EUR dataset. Cox Regression models with Firth’s Penalized Likelihood, as implemented in the R *coxphf* package (v1.13.4) and function, were used to compare disease risk according to sex across PD cases and non-neurological controls from the UKB EUR, AMP-PD 100kGP, and AoU cohorts. Sex was modeled as a binary variable with Female as the reference category. We included cohort as a covariate to control for this possible confound, comparing the AMP-PD 100kGP, and AoU cohorts against the UKB EUR reference category. Kaplan-Meier curves were generated with the R packages ggplot2 (v3.5.1), ggsurvfit (v1.1.0), and patchwork (v1.3.0).

#### Clinical case series

For the 780 BCM cases, DNA was extracted from peripheral blood lymphocytes samples using the E.Z.N.A. SQ Blood DNA Kit (Omega Bio-tek Inc.), or from saliva samples using the Gentra Puregene Blood Kit (DNA purification from body fluid protocol) (QIAGEN). Whole-exome sequencing was performed by Regeneron Genetics Center (RGC). The samples were processed with the Twist Comprehensive Exome design combined with RGC-designed spike-ins for sequencing genotyping sites, the full mitochondrial genome, and boosting coverage at selected sites for assaying clonal hematopoiesis of indeterminant potential (CHIP), and sequenced on the Illumina NovaSeq 6000 system with 75-bp paired-end reads and two index reads.

All variants, including both exonic and intronic, were annotated using the Ensembl Variant Effect Predictor (v 113). We filtered variants to those with a gnomAD popmax MAF<0.1% and considered PTVs as those with the following annotations: exon_loss_variant, frameshift_variant, start_lost, stop_gained, stop_lost, splice_acceptor_variant, splice_donor_variant, gene_fusion, bidirectional_gene_fusion, rare_amino_acid_variant, and transcript_ablation. We observed a total of three PTVs, all of which were singletons, occurred in individuals of EUR ancestry (predicted by Peddy^65^), and were validated via Sanger sequencing.

1. 21–33794425-C/T (stop gain)
2. 21–33782020-GC/G (frameshift)
3. 21–33750248-C-CCA (frameshift)

#### *Drosophila* experiments

##### Motor performance assays

Age-dependent motor performance was assessed using ten age-matched females per-replica per-genotype as previously described.^57,58^ Females were collected over a 24-h period and transferred into new media daily. Animals were kept at 25°C. Using an automated platform, flies were tapped to the bottom of a plastic vial and recorded for 7.5s as they climbed back up. Videos were analyzed using custom software to assess the speed and stumbling events of each animal. Four trials were performed each day per replicate, with four replicates per genotype. Data were analyzed using a nonlinear random mixed effect model ANOVA applied to spline regressions, adjusting *p*-values for multiplicity using Holm’s procedure. All graphing and statistical analyses were performed in R. Negative controls with baseline disease and motor performance were established using a neutral UAS line.

##### Eye imaging

Fruit flies were raised at 29°C. Eye images were captured from adult (1-day old) female flies using the Leica MZ16 imaging system. Areas of the eye showing fused or missing facets were selected with ImageJ. The average ratio of “disorganized eye area/total eye area” was calculated for α-synuclein-expressing fly genotypes. Data was analyzed using ANOVA followed by Dunnet’s post hoc test comparing to the α-Syn*/no modifier* genotype.

##### Neuromuscular junction immunofluorescence

Larvae were raised at 28°C. Third-instar larvae were dissected in cold PBS and fixed (diH2O, 10% formaldehyde, 10X PBS at a 5:4:1 ratio) for 30 min. After 1hr blocking, larva fillets were incubated in primary antibodies on a shaker at 4°C for 2 days (BD Transduction Laboratories mouse anti-α-synuclein Cat. #610787 1:200, chicken anti-Dap160^70^ 1:500), rabbit anti-HRP (Jackson ImmunoResearch Ref. #323–005-021). Secondaries were obtained from ThermoFisher and used at 1:400 (Alexa Fluor 647 goat antimouse IgG Cat. #A32728, Alexa Fluor 488 goat anti-chicken IgY Cat. #A-11039, Alexa Fluor 555 goat anti-rabbit IgG Cat. #A32732) and 1:1000 (DAPI Cat. #62248). Larval fillets were mounted using invitrogen ProLong Glass Antifade Mountant (Ref. #P36980) and imaged using a Leica SP8 confocal microscope. Colocalization was analyzed on single slices and quantified using ImageJ.

#### HEK293 co-localization experiments

##### Cloning and mutagenesis

We obtained ITSN1-FLAG construct from Addgene (RRID: Addgene_47393).^71^ We added C-terminal V5-tag to SNCA in PCDNA6 vector (RRID: Addgene_107425)^72^ using the NEB Q5 site-directed mutagenesis kit (catalog #E0554S). We used 5′-ctgctgggcctggatagcaccTAAGAAATATCTTTGCTCCC-3′ for the forward primer and 5′-cgggttcggaatcggtttgccGGCTTCAGGTTCGTAG-3′ as our reverse primer. We performed the mutagenesis reaction according to manufacturer instructions with an annealing temperature of 57C and elongation time of 2 min and 20 s. We transformed the plasmids into NEBAlpha (NEB) cells and verified tag insertion by whole-plasmid sequencing (Azenta).

##### Cell culture and transfection

HEK293T cells were transfected with plasmids encoding cDNA TransIT-293 transfection reagent, according to the manufacturer’s protocol, and incubated for 48–72 h. 2μg of ITNS1-FLAG and 0.5mg of SNCA-V5 or non-inserted V5 (control) transfected to one of 6 wells. After 24–48 h of infection, cells were incubated with the appropriate antibiotics for at least 72 h. Cells were further maintained until additional treatment or collection.

##### Cell lysis

Frozen HEK293T cells in plates were briefly thawed on ice and lysed by gentle mixing with ice-cold lysis buffer (50 mM Tris, 150 mM NaCl, 2 mM CaCl_2_, 0.5% Triton X-100, 1% NP-40, 5 mM EDTA, 5% glycerol, pH 7.5) containing 1× protease and phosphatase inhibitor cocktails (Genedepot, P3100–100, P3200–020) on a shaker for 30–60 min at 4°C. The cell supernatant was collected by centrifugation at 15,000 rpm for 30 min and subjected to further experiments. The total protein concentration of the brain tissue and cell lysate was determined using a BCA Protein Assay Kit (Thermo Scientific, 10–009-D).

##### Immunoprecipitation

Supernatant from one well of a 6-well plate was incubated with 1 μg of anti-FLAG antibody (RRID: AB_262044) conjugated with protein G Dynabeads (15 μL slurry, Fisher Scientific, 10–009-D) for 2–4 h at 4°C. After washing with lysis buffer, immunoprecipitated proteins were eluted in 35 μL of 1.53 Laemmli sample buffer (Sigma-Aldrich, S3401–10VL) by boiling at 75°C for 10 min.

##### SDS-PAGE and western blot

Protein samples were loaded onto 10-well NuPAGE 4–12% Bis-Tris gels (Thermo Fisher Scientific). Gels were run in 1× MES/SDS protein running buffer and transferred onto nitrocellulose membranes in Tris-Glycine buffer (25 mM Tris, 190 mM glycine) supplemented with 10% methanol at 0.3 amps for 1.5 h. After transfer, membranes were blocked in 5% milk in TBS-T for 1 h and probed with one of the following primary antibodies overnight: mouse anti-FLAG (1:10,000) or mouse anti-α-Syn (BD Bioscience, RRID: AB_398107, 1:3,000). Membranes were washed three times in TBS-T for 10 min, and HRP-conjugated secondary antibodies were applied in 5% skim milk in TBS-T. Following additional washes, chemiluminescence was induced using ECL (Cytiva, RPN2236) and imaged with an Amersham Imager 680 (GE Healthcare).

### QUANTIFICATION AND STATISTICAL ANALYSIS

All statistical analyses was performed using PEACOK v1.07, R v v4.1.0, and PLINK 2.0. All statistical tests are named as they are used in figure legends and results, and all described in the method details.

#### Genomic inflation and p Value thresholds

Lambda was estimated using a previously published approach, in which an empirical null *p*-value distribution was generated using an n-of-1 permutation of case-control labels.^12^ Consistent with previous studies, discovery analyses were interpreted against a conservative significance threshold of p < 1 × 10^−8^.^69^ This threshold was determined on the evaluation of the smallest *p*-values from the n-of-1 permutation (collapsing analysis *p*_min_ = 3.5 × 10^−6^, ExWAS *p*_min_ = 5.4 × 10^−7^) tests and from the synonymous collapsing analysis model (*p*_min_ = 4.2 × 10^−5^). We chose p < 1 × 10^−6^ as the “suggestive” threshold to identify signals that warranted follow-up via replication analyses. Replication analyses were conducted with a nominal significance threshold of *p* < 0.05.

#### Power analysis

Power analysis was performed to assess the sample requirements for 80% power to replicate the association between PD and *ITSN1* PTVs at *p* = 0.05. Assuming a cumulative minor allele frequency of approximately 0.0003 for *ITSN1* PTVs (Tables S9 and S10), we estimated that a total of ~17,500 cases and controls (at 1:1 sampling) would be required to detect an association with an odds ratio of 10, and ~25,000 for an odds ratio of 6 (Figure S5).

## Supplementary Material

1

10

11

12

2

3

4

5

6

7

8

9

## Figures and Tables

**Table 1.** Top-ranking gene-level associations from the collapsing analysis

Gene symbol	*p*	Odds ratio [95% confidence interval]	No. of individuals with qualifying variant/total no. of individuals		Model with most significant association (rank in model)
			Cases	Controls	

*GBA1*	2.41 × 10^−9^	3.54 [2.49, 5.04]	33/3,702	591/233,378	flexnonsynmtr (1)
*ITSN1*	6.05 × 10^−7^	10.53 [5.2, 21.34]	9/3,702	54/233,378	ptv (1)
*ADH5*	2.71 × 10^−5^	2.57 [1.74, 3.81]	26/3,702	640/233,378	flexdmg (2)
*MERTK*	4.17 × 10^−5^	2.83 [1.83, 4.39]	21/3,702	469/233,378	raredmgmtr (1)
*PLEKHB2*	4.70 × 10^−5^	8.19 [3.72, 18]	7/3,702	54/233,378	UR (1)
*ATP6V1B2*	5.62 × 10^−5^	0.093 [0.002, 0.52]^a^	0/3,702	671/233,378	flexdmg (3)
*NEK11*	6.89 × 10^−5^	1.99 [1.46, 2.71]	42/3,702	1,338/233,378	flexnonsynmtr (2)
*ZNF554*	7.82 × 10^−5^	0.24 [0.1, 0.58]	5/3,702	1,311/233,378	flexnonsynmtr (3)

Included are all gene-level associations that achieved *p* <1 × 10^−4^ across the nine non-synonymous collapsing models. The study-wide significance threshold is *p* < 1 × 10^−8^. *p* values were calculated via a two-tailed Fisher’s exact test.

a Due to the absence of QVs in cases, we adjusted the contingency table by adding one to all cells to calculate an odds ratio.

**Table:** KEY RESOURCES TABLE

REAGENT or RESOURCE	SOURCE	IDENTIFIER

Antibodies		

Anti-Alpha-Synuclein antibody (clone 42) (1:200)	BD Transduction Laboratories	RRID: AB_398107
Anti-ITSN1	Abcam	RRID: AB_10899433
Monoclonal ANTI-FLAG ^®^ M2 antibody produced in mouse	SIGMA-ALDRICH	RRID: AB_262044
Chicken anti-Dap160 (1:500)	Chris Q. Doe (Chabu and Doe)^47^	N/A
Rabbit anti-HRP (1:200)	Jackson ImmunoResearch	RRID: AB_2314648
Alexa Fluor 647 Goat anti-Mouse IgG (1:400)	ThermoFisher	RRID: AB_2633277
Alexa Fluor 488 Goat anti-Chicken IgY (1:400)	ThermoFisher	RRID: AB_2534096
Alexa Fluor Goat anti-Rabbit IgG (1:400)	ThermoFisher	RRID: AB_2633281

Chemicals, peptides, and recombinant proteins		

DAPI (1:1000)	ThermoFisher	Cat. #62248
Dynabeads Protein G	Thermo Fisher	Cat. #10009D
1× protease inhibitor cocktails	GeneDepot	P3100–100
1× phosphatase inhibitor cocktails	GeneDepot	P3200–020
Laemmli sample buffer	SIGMA-ALDRICH	Cat. #S3401–10VL
Amersham ECL Prime Western Blotting	Cytiva	RPN2236
Detection Reagent		
TransIT-293 transfection reagent	Mirus Bio	MIR2704

Critical commercial assays		

NEB Q5 site-directed mutagenesis kit	New England Biolabs	Cat. #E0554S

Deposited data		

Full ExWAS and collapsing analysis summary statistics	This paper	https://doi.org/10.5281/zenodo.14705135

Experimental models: Cell lines		

HEK293T cells	ATCC	RRID: CVCL_0063

Experimental models: Organisms/strains		

*Drosophila*/w[118]; P{w[+mC] = UAS-SNCA.J}1/Cyo	Bloomington Drosophila Stock Center (BDSC)	51375
*Drosophila*/P{w[+mW.hs] = GawB}elav[C155]	BDSC	458
*Drosophila*/P{GAL4-ninaE.GMR}12	BDSC	1104
*Drosophila*/Dap160Δ1	BDSC	24877
*Drosophila*/Dap160Δ2	BDSC	24878
*Drosophila*/P{EP}Dap160[EP2543]	BDSC	19582
*Drosophila*/P{w[+mC] = UAS-Dap160.K}4.1	BDSC	42695

Oligonucleotides		

For SNCA-V5 cloning Forward primer: 5′-ctgctgggcctggatagcacc TAAGAAATATCTTTGCTCCC-3′	Sigma-Aldrich	N/A
For SNCA-V5 cloning Reverse primer 5′-cgggttcggaatcggtttgcc GGCTTCAGGTTCGTAG-3′	Sigma-Aldrich	N/A

Recombinant DNA		

ITSN1-FLAG	Addgene	RRID: Addgene_47393
PCDNA6-V5	Addgene	RRID: Addgene_107425
PCDNA6-SNCA-V5	This paper	N/A

Software and algorithms		

SnpEff v4.3	Cingolani et al.^48^	https://pcingola.github.io/SnpEff/
Variant Effect Predictor v103	McLaren et al.^49^	https://www.ensembl.org/vep
Python 3.10.12	Python Software Foundation	https://www.python.org
R v4.1.0 with packages stats v4.1.0, UKBtools v0.11.3, logistf v1.26.0, survival v3.7.0, coxphf v1.13.4, ggplot2 v3.5.1, ggsurvfit v1.1.0, and patchwork v1.3.0.	R Foundation	https://www.r-project.org/
KING relatedness v2.2.3	Manichaikul et al.^50^	https://www.kingrelatedness.com/
PLINK2 v5.10	Chang et al.^51^	https://www.cog-genomics.org/plink/2.0/
Peddy v0.4.2	Pedersen et al.^52^	https://github.com/brentp/peddy
PEACOK v1.07	Wang et al.^8^	https://doi.org/10.5281/zenodo.7097303

Other		

Clinical and WGS data from the UK Biobank	Li et al.^48^	https://www.ukbiobank.ac.uk/
Demographics and short read whole-genome sequence data from All of Us (v7)	Bick et al.^53^	https://allofus.nih.gov/
Clinical and joint-genotyping sequence data from AMP-PD (v4)	Iwaki et al.^17^	https://doi.org/10.1038/s41586-023-06957-x
Clinical and WGS data from the 100,000 Genomes Project	Caulfield et al.^54^	https://re-docs.genomicsengland.co.uk/
Summary data from deCODE genetics cohort	Skuladottir et al.^7^	https://doi.org/10.1038/s41531-024-00752-9
Gene expression data from GTEx	Lonsdale et al.^15^	https://www.gtexportal.org/home/
Protein abundance data from a subset of GTEx cohort	Jiang et al.^16^	https://doi.org/10.1016/j.cell.2020.08.036
Consensus CDS (CCDS) database release 22	Pujar et al.^49^	https://doi.org/10.1093/nar/gkx1031
Variant frequency and quality control information from gnomAD v2.1.1.	Karczewski et al.^55^	https://gnomad.broadinstitute.org/

## Highlights

- *ITSN1* haploinsufficiency confers a ~10-fold increased risk of Parkinson’s disease (PD)
- Effect size surpasseas other well-established loci, including *GBA1* and *LRRK2*
- *In vivo* and *in vitro* studies suggest an interaction between ITSN1 α-synuclein
- Findings implicate synaptic vesicle trafficking dysfunction in PD pathogenesis

## References

[1] Ye H, Robak LA, Yu M, Cykowski M, and Shulman JM (2023). Genetics and Pathogenesis of Parkinson’s Syndrome. Annu. Rev. Pathol 18, 95–121. 10.1146/annurev-pathmechdis-031521-034145. 36100231 PMC10290758

[2] Cheng H-C, Ulane CM, and Burke RE (2010). Clinical progression in Parkinson disease and the neurobiology of axons. Ann. Neurol 67, 715–725. 10.1002/ana.21995. 20517933 PMC2918373

[3] Bloem BR, Okun MS, and Klein C. (2021). Parkinson’s disease. Lancet Lond. Engl 397, 2284–2303. 10.1016/S0140-6736(21)00218-X. 33848468

[4] Blauwendraat C, Nalls MA, and Singleton AB (2020). The genetic architecture of Parkinson’s disease. Lancet Neurol. 19, 170–178. 10.1016/S1474-4422(19)30287-X. 31521533 PMC8972299

[5] Nalls MA, Blauwendraat C, Vallerga CL, Heilbron K, Bandres-Ciga S, Chang D, Tan M, Kia DA, Noyce AJ, Xue A, (2019). Identification of novel risk loci, causal insights, and heritable risk for Parkinson’s disease: a meta-genome wide association study. Lancet Neurol. 18, 1091–1102. 10.1016/S1474-4422(19)30320-5. 31701892 PMC8422160

[6] Makarious MB, Lake J, Pitz V, Ye Fu A, Guidubaldi JL, Solsberg CW, Bandres-Ciga S, Leonard HL, Kim JJ, Billingsley KJ, (2023). Large-scale rare variant burden testing in Parkinson’s disease. Brain 146, 4622–4632. 10.1093/brain/awad214. 37348876 PMC10629770

[7] Skuladottir AT, Tragante V, Sveinbjornsson G, Helgason H, Sturluson A, Bjornsdottir A, Jonsson P, Palmadottir V, Sveinsson OA, Jensson BO, (2024). Loss-of-function variants in ITSN1 confer high risk of Parkinson’s disease. NPJ Parkinsons Dis. 10, 140. 10.1038/s41531-024-00752-9. 39147844 PMC11327306

[8] Wang Q, Dhindsa RS, Carss K, Harper AR, Nag A, Tachmazidou I, Vitsios D, Deevi SVV, Mackay A, Muthas D, (2021). Rare variant contribution to human disease in 281,104 UK Biobank exomes. Nature 597, 527–532. 10.1038/s41586-021-03855-y. 34375979 PMC8458098

[9] Van Den Eeden SK, Tanner CM, Bernstein AL, Fross RD, Leimpeter A, Bloch DA, and Nelson LM (2003). Incidence of Parkinson’s disease: variation by age, gender, and race/ethnicity. Am. J. Epidemiol 157, 1015–1022. 10.1093/aje/kwg068. 12777365

[10] Prahl J, and Coetzee GA (2022). Genetic Elements at the Alpha-Synuclein Locus. Front. Neurosci 16, 889802. 10.3389/fnins.2022.889802. 35898413 PMC9309432

[11] Smith L, and Schapira AHV (2022). GBA Variants and Parkinson Disease: Mechanisms and Treatments. Cells 11, 1261. 10.3390/cells11081261. 35455941 PMC9029385

[12] Petrovski S, Todd JL, Durheim MT, Wang Q, Chien JW, Kelly FL, Frankel C, Mebane CM, Ren Z, Bridgers J, (2017). An Exome Sequencing Study to Assess the Role of Rare Genetic Variation in Pulmonary Fibrosis. Am. J. Respir. Crit. Care Med. 196, 82–93. 10.1164/rccm.201610-2088OC. 28099038 PMC5519963

[13] Dhindsa RS, Burren OS, Sun BB, Prins BP, Matelska D, Wheeler E, Mitchell J, Oerton E, Hristova VA, Smith KR, (2023). Rare variant associations with plasma protein levels in the UK Biobank. Nature 622, 339–347. 10.1038/s41586-023-06547-x. 37794183 PMC10567546

[14] Dorion M-F, Yaqubi M, Senkevich K, Kieran NW, MacDonald A, Chen CXQ, Luo W, Wallis A, Shlaifer I, Hall JA, (2024). MerTK is a mediator of alpha-synuclein fibril uptake by human microglia. Brain 147, 427–443. 10.1093/brain/awad298. 37671615 PMC10834256

[15] Lonsdale J, Thomas J, Salvatore M, Phillips R, Lo E, Shad S, Hasz R, Walters G, Garcia F, Young N, (2013). The Genotype-Tissue Expression (GTEx) project. Nat. Genet 45, 580–585. 10.1038/ng.2653. 23715323 PMC4010069

[16] Jiang L, Wang M, Lin S, Jian R, Li X, Chan J, Dong G, Fang H, Robinson AE, and GTEx Consortium; and Snyder MP (2020). A Quantitative Proteome Map of the Human Body. Cell 183, 269–283.e19. 10.1016/j.cell.2020.08.036. 32916130 PMC7575058

[17] Zhou X, Feliciano P, Shu C, Wang T, Astrovskaya I, Hall JB, Obiajulu JU, Wright JR, Murali SC, Xu SX, (2022). Integrating de novo and inherited variants in 42,607 autism cases identifies mutations in new moderate-risk genes. Nat. Genet 54, 1305–1319. 10.1038/s41588-022-01148-2. 35982159 PMC9470534

[18] Ali A, Milman S, Weiss EF, Gao T, Napolioni V, Barzilai N, Zhang ZD, and Lin J-R (2025). Genetic variants associated with age-related episodic memory decline implicate distinct memory pathologies. Alzheimers Dement. 21, e14379. 10.1002/alz.14379. 39559945 PMC11775541

[19] Iwaki H, Leonard HL, Makarious MB, Bookman M, Landin B, Vismer D, Casey B, Gibbs JR, Hernandez DG, Blauwendraat C, (2021). Accelerating Medicines Partnership: Parkinson’s Disease. Genetic Resource. Mov. Disord 36, 1795–1804. 10.1002/mds.28549. 33960523 PMC8453903

[20] Ortega RA, Bressman SB, Raymond D, Ozelius LJ, Katsnelson V, Leaver K, Swan MC, Shanker V, Miravite J, Wang C, (2022). Differences in Sex-Specific Frequency of Glucocerebrosidase Variant Carriers and Familial Parkinsonism. Mov. Disord 37, 2217–2225. 10.1002/mds.29197. 36054306 PMC9669136

[21] Chen W, Yan X, Lv H, Liu Y, He Z, and Luo X. (2020). Gender differences in prevalence of LRRK2-associated Parkinson disease: A meta-analysis of observational studies. Neurosci. Lett 715, 134609. 10.1016/j.neulet.2019.134609. 31698024

[22] Li Q, Jing Y, Lun P, Liu X, and Sun P. (2021). Association of gender and age at onset with glucocerebrosidase associated Parkinson’s disease: a systematic review and meta-analysis. Neurol. Sci 42, 2261–2271. 10.1007/s10072-021-05230-1. 33837876

[23] Orchard S, Ammari M, Aranda B, Breuza L, Briganti L, Broackes-Carter F, Campbell NH, Chavali G, Chen C, del-Toro N, (2014). The MIntAct project−IntAct as a common curation platform for 11 molecular interaction databases. Nucleic Acids Res. 42, D358–D363. 10.1093/nar/gkt1115. 24234451 PMC3965093

[24] Jin M, Iwamoto Y, Shirazinejad C, and Drubin DG (2024). Intersectin1 promotes clathrin-mediated endocytosis by organizing and stabilizing endocytic protein interaction networks. Preprint at bioRxiv, 2024.04.22. 590579. 10.1101/2024.04.22.590579. PMC11728081 39580802

[25] Stenson PD, Ball EV, Mort M, Phillips AD, Shiel JA, Thomas NST, Abeysinghe S, Krawczak M, and Cooper DN (2003). Human Gene Mutation Database (HGMD): 2003 update. Hum. Mutat 21, 577–581. 10.1002/humu.10212. 12754702

[26] Middleton L, Melas I, Vasavda C, Raies A, Rozemberczki B, Dhindsa RS, Dhindsa JS, Weido B, Wang Q, Harper AR, (2024). Phenome-wide identification of therapeutic genetic targets, leveraging knowledge graphs, graph neural networks, and UK Biobank data. Sci. Adv 10, eadj1424. 10.1126/sciadv.adj1424. 38718126 PMC11078195

[27] Li J, Amoh BK, McCormick E, Tarkunde A, Zhu KF, Perez A, Mair M, Moore J, Shulman JM, Al-Ramahi I, and Botas J. (2023). Integration of transcriptome-wide association study with neuronal dysfunction assays provides functional genomics evidence for Parkinson’s disease genes. Hum. Mol. Genet 32, 685–695. 10.1093/hmg/ddac230. 36173927 PMC9896475

[28] Yu M, Ye H, De-Paula RB, Mangleburg CG, Wu T, Lee TV, Li Y, Duong D, Phillips B, Cruchaga C, (2023). Functional screening of lysosomal storage disorder genes identifies modifiers of alpha-synuclein neurotoxicity. PLoS Genet. 19, e1010760. 10.1371/journal.pgen.1010760. 37200393 PMC10231792

[29] Hirth F. (2010). Drosophila melanogaster in the study of human neurodegeneration. CNS Neurol. Disord.: Drug Targets 9, 504–523. 10.2174/187152710791556104. 20522007 PMC2992341

[30] Matsuoka T, Yoshida H, Kasai T, Tozawa T, Iehara T, and Chiyonobu T. (2024). α-synuclein pathology in Drosophila melanogaster is exacerbated by haploinsufficiency of Rop: connecting STXBP1 encephalopathy with α-synucleinopathies. Hum. Mol. Genet 33, 1328–1338. 10.1093/hmg/ddae073. 38692286

[31] Koh T-W, Verstreken P, and Bellen HJ (2004). Dap160/intersectin acts as a stabilizing scaffold required for synaptic development and vesicle endocytosis. Neuron 43, 193–205. 10.1016/j.neuron.2004.06.029. 15260956

[32] Marie B, Sweeney ST, Poskanzer KE, Roos J, Kelly RB, and Davis GW (2004). Dap160/intersectin scaffolds the periactive zone to achieve high-fidelity endocytosis and normal synaptic growth. Neuron 43, 207–219. 10.1016/j.neuron.2004.07.001. 15260957

[33] Groza T, Gomez FL, Mashhadi HH, Muñoz-Fuentes V, Gunes O, Wilson R, Cacheiro P, Frost A, Keskivali-Bond P, Vardal B, (2023). The International Mouse Phenotyping Consortium: comprehensive knockout phenotyping underpinning the study of human disease. Nucleic Acids Res. 51, D1038–D1045. 10.1093/nar/gkac972. 36305825 PMC9825559

[34] Gustavsson EK, Follett J, Trinh J, Barodia SK, Real R, Liu Z, Grant-Peters M, Fox JD, Appel-Cresswell S, Stoessl AJ, (2024). RAB32 Ser71Arg in autosomal dominant Parkinson’s disease: linkage, association, and functional analyses. Lancet Neurol. 23, 603–614. 10.1016/S1474-4422(24)00121-2. 38614108 PMC11096864

[35] Hop PJ, Lai D, Keagle PJ, Baron DM, Kenna BJ, Kooyman M, and Asselta R. (2024). Systematic rare variant analyses identify RAB32 as a susceptibility gene for familial Parkinson’s disease. Nat. Genet 56, 1371–1376. 10.1038/s41588-024-01787-7. 38858457 PMC11250361

[36] Szklarczyk D, Kirsch R, Koutrouli M, Nastou K, Mehryary F, Hachilif R, Gable AL, Fang T, Doncheva NT, Pyysalo S, (2023). The STRING database in 2023: protein-protein association networks and functional enrichment analyses for any sequenced genome of interest. Nucleic Acids Res. 51, D638–D646. 10.1093/nar/gkac1000. 36370105 PMC9825434

[37] Erb ML, and Moore DJ (2020). LRRK2 and the Endolysosomal System in Parkinson’s Disease. J. Parkinsons Dis. 10, 1271–1291. 10.3233/JPD-202138. 33044192 PMC7677880

[38] Pan P-Y, Sheehan P, Wang Q, Zhu X, Zhang Y, Choi I, Li X, Saenz J, Zhu J, Wang J, (2020). Synj1 haploinsufficiency causes dopamine neuron vulnerability and alpha-synuclein accumulation in mice. Hum. Mol. Genet 29, 2300–2312. 10.1093/hmg/ddaa080. 32356558 PMC7424763

[39] Soukup S-F, Vanhauwaert R, and Verstreken P. (2018). Parkinson’s disease: convergence on synaptic homeostasis. EMBO J. 37, e98960. 10.15252/embj.201898960. 30065071 PMC6138432

[40] Yu Y, Chu P-Y, Bowser DN, Keating DJ, Dubach D, Harper I, Tkalcevic J, Finkelstein DI, and Pritchard MA (2008). Mice deficient for the chromosome 21 ortholog Itsn1 exhibit vesicle-trafficking abnormalities. Hum. Mol. Genet 17, 3281–3290. 10.1093/hmg/ddn224. 18676989

[41] Starkstein S, Gellar S, Parlier M, Payne L, and Piven J. (2015). High rates of parkinsonism in adults with autism. J. Neurodev. Disord 7, 29. 10.1186/s11689-015-9125-6. 26322138 PMC4553212

[42] Dhindsa RS, Bradrick SS, Yao X, Heinzen EL, Petrovski S, Krueger BJ, Johnson MR, Frankel WN, Petrou S, Boumil RM, and Goldstein DB (2015). Epileptic encephalopathy-causing mutations in DNM1 impair synaptic vesicle endocytosis. Neurol. Genet 1, e4. 10.1212/01.NXG.0000464295.65736.da. 27066543 PMC4821085

[43] De Rubeis S, He X, Goldberg AP, Poultney CS, Samocha K, Cicek AE, Kou Y, Liu L, Fromer M, Walker S, (2014). Synaptic, transcriptional and chromatin genes disrupted in autism. Nature 515, 209–215. 10.1038/nature13772. 25363760 PMC4402723

[44] Rizig M, Bandres-Ciga S, Makarious MB, Ojo OO, Crea PW, Abiodun OV, Levine KS, Abubakar SA, Achoru CO, Vitale D, (2023). Identification of genetic risk loci and causal insights associated with Parkinson’s disease in African and African admixed populations: a genome-wide association study. Lancet Neurol. 22, 1015–1025. 10.1016/S1474-4422(23)00283-1. 37633302 PMC10593199

[45] Wang Q. (2020). Acoustic energy isotherms: An emergent approach for textural characterization of activated carbons. Microporous and Mesoporous Materials 298, 110045. 10.5281/zenodo.7097303.

[46] Spargo T, and Dhindsa R. (2025). Haploinsufficiency of ITSN1 is associated with a substantial increased risk of Parkinson’s disease - summary statistics. 10.5281/zenodo.14705135. PMC12124131 40056900

[47] Chang CC, Chow CC, Tellier LC, Vattikuti S, Purcell SM, and Lee JJ (2015). Second-generation PLINK: rising to the challenge of larger and richer datasets. GigaScience 4, 7. 10.1186/s13742-015-0047-8. 25722852 PMC4342193

[48] Li S, Carss KJ, Halldorsson BV, and Cortes A; UK Biobank Whole-Genome Sequencing Consortium (2023). Whole-genome sequencing of half-a-million UK Biobank participants. Preprint at medRxiv. 10.1101/2023.12.06.23299426.

[49] Pujar S, O’Leary NA, Farrell CM, Loveland JE, Mudge JM, Wallin C, Girón CG, Diekhans M, Barnes I, Bennett R, (2018). Consensus coding sequence (CCDS) database: a standardized set of human and mouse protein-coding regions supported by expert curation. Nucleic Acids Res. 46, D221–D228. 10.1093/nar/gkx1031. 29126148 PMC5753299

[50] Caulfield M, Davies J, Dennys M, Elbahy L, Fowler T, Hill S, Hubbard T, Jostins L, Maltby N, Mahon-Pearson J, (2020). Database resources of the national genomics data center in 2020. Nucleic acids research 48, D24–D33. 10.6084/m9.figshare.4530893.v7. 31702008 PMC7145560

[51] Nalls MA, Bras J, Hernandez DG, Keller MF, Majounie E, Renton AE, Saad M, Jansen I, Guerreiro R, Lubbe S, (2015). NeuroX, a fast and efficient genotyping platform for investigation of neurodegenerative diseases1605.e7–1605.e1.605E12. Neurobiol. Aging 36. 10.1016/j.neurobiolaging.2014.07.028. PMC4317375 25444595

[52] Jun G, Flickinger M, Hetrick KN, Romm JM, Doheny KF, Abecasis GR, Boehnke M, and Kang HM (2012). Detecting and estimating contamination of human DNA samples in sequencing and array-based genotype data. Am. J. Hum. Genet 91, 839–848. 10.1016/j.ajhg.2012.09.004. 23103226 PMC3487130

[53] Bick AG, Metcalf GA, Mayo KR, Lichtenstein L, Rura S, Carroll RJ, Musick A, Linder JE, Jordan IK, Nagar SD, (2024). Genomic data in the All of Us Research Program. Nature 627, 340–346. 10.1038/s41586-023-06957-x. 38374255 PMC10937371

[54] 100000 Genomes Project Pilot Investigators; Smedley D, Smith KR, Martin A, Thomas EA, McDonagh EM, Cipriani V, Ellingford JM, Arno G, and Tucci A. (2021). 100,000 Genomes Pilot on Rare-Disease Diagnosis in Health Care - Preliminary Report. N. Engl. J. Med 385, 1868–1880. 10.1056/NEJMoa2035790. 34758253 PMC7613219

[55] Karczewski KJ, Francioli LC, Tiao G, Cummings BB, Alföldi J, Wang Q, Collins RL, Laricchia KM, Ganna A, Birnbaum DP, (2020). The mutational constraint spectrum quantified from variation in 141,456 humans. Nature 581, 434–443. 10.1038/s41586-020-2308-7. 32461654 PMC7334197

[56] Robak LA, Du R, Yuan B, Gu S, Alfradique-Dunham I, Kondapalli V, Hinojosa E, Stillwell A, Young E, Zhang C, (2020). Integrated sequencing and array comparative genomic hybridization in familial Parkinson disease. Neurol. Genet 6, e498. 10.1212/NXG.0000000000000498. 32802956 PMC7413630

[57] Rousseaux MWC, Vázquez-Vélez GE, Al-Ramahi I, Jeong H-H, Bajić A, Revelli J-P, Ye H, Phan ET, Deger JM, Perez AM, (2018). A Druggable Genome Screen Identifies Modifiers of α-Synuclein Levels via a Tiered Cross-Species Validation Approach. J. Neurosci. Off. J. Soc. Neurosci 38, 9286–9301. 10.1523/JNEUROSCI.0254-18.2018. PMC6199406 30249792

[58] Onur TS, Laitman A, Zhao H, Keyho R, Kim H, Wang J, Mair M, Wang H, Li L, Perez A, (2021). Downregulation of glial genes involved in synaptic function mitigates Huntington’s disease pathogenesis. Elife 10, e64564. 10.7554/eLife.64564. 33871358 PMC8149125

[59] Halldorsson BV, Eggertsson HP, Moore KHS, Hauswedell H, Eiriksson O, Ulfarsson MO, Palsson G, Hardarson MT, Oddsson A, Jensson BO, (2022). The sequences of 150,119 genomes in the UK Biobank. Nature 607, 732–740. 10.1038/s41586-022-04965-x. 35859178 PMC9329122

[60] Cingolani P, Platts A, Wang LL, Coon M, Nguyen T, Wang L, Land SJ, Lu X, and Ruden DM (2012). A program for annotating and predicting the effects of single nucleotide polymorphisms, SnpEff: SNPs in the genome of Drosophila melanogaster strain w1118; iso-2; iso-3. Fly 6, 80–92. 10.4161/fly.19695. 22728672 PMC3679285

[61] Howe KL, Achuthan P, Allen J, Allen J, Alvarez-Jarreta J, Amode MR, Armean IM, Azov AG, Bennett R, Bhai J, (2021). Ensembl 2021. Nucleic Acids Res. 49, D884–D891. 10.1093/nar/gkaa942. 33137190 PMC7778975

[62] Manichaikul A, Mychaleckyj JC, Rich SS, Daly K, Sale M, and Chen W-M (2010). Robust relationship inference in genome-wide association studies. Bioinformatics 26, 2867–2873. 10.1093/bioinformatics/btq559. 20926424 PMC3025716

[63] Hanscombe KB, Coleman JRI, Traylor M, and Lewis CM (2019). ukbtools: An R package to manage and query UK Biobank data. PLoS One 14, e0214311. 10.1371/journal.pone.0214311. 31150407 PMC6544205

[64] 1000 Genomes Project Consortium; Auton A, Brooks LD, Durbin RM, Garrison EP, Kang HM, Korbel JO, Marchini JL, McCarthy S, McVean GA, and Abecasis GR. (2015). A global reference for human genetic variation. Nature 526, 68–74. 10.1038/nature15393. 26432245 PMC4750478

[65] Pedersen BS, and Quinlan AR (2017). Who’s Who? Detecting and Resolving Sample Anomalies in Human DNA Sequencing Studies with Peddy. Am. J. Hum. Genet 100, 406–413. 10.1016/j.ajhg.2017.01.017. 28190455 PMC5339084

[66] McLaren W, Gil L, Hunt SE, Riat HS, Ritchie GRS, Thormann A, Flicek P, and Cunningham F. (2016). The Ensembl Variant Effect Predictor. Genome Biol. 17, 122. 10.1186/s13059-016-0974-4. 27268795 PMC4893825

[67] International HapMap 3 Consortium; Altshuler DM, Altshuler DM, Peltonen L, Gibbs RA, Peltonen L, Peltonen L, Dermitzakis E, Schaffner SF, Peltonen L, and Yu F. (2010). Integrating common and rare genetic variation in diverse human populations. Nature 467, 52–58. 10.1038/nature09298. 20811451 PMC3173859

[68] Rimmer A, Phan H, Mathieson I, Iqbal Z, Twigg SRF, WGS500 Consortium; Wilkie AOM, McVean G, and Lunter G. (2014). Integrating mapping-assembly- and haplotype-based approaches for calling variants in clinical sequencing applications. Nat. Genet 46, 912–918. 10.1038/ng.3036. 25017105 PMC4753679

[69] Nag A, Dhindsa RS, Mitchell J, Vasavda C, Harper AR, Vitsios D, Ahnmark A, Bilican B, Madeyski-Bengtson K, Zarrouki B, (2022). Human genetics uncovers MAP3K15 as an obesity-independent therapeutic target for diabetes. Sci. Adv 8, eadd5430. 10.1126/sciadv.add5430. 36383675 PMC9668288

[70] Chabu C, and Doe CQ (2008). Dap160/intersectin binds and activates aPKC to regulate cell polarity and cell cycle progression. Dev. Camb. Engl 135, 2739–2746. 10.1242/dev.024059. PMC2683763 18614576

[71] Hussain NK, Jenna S, Glogauer M, Quinn CC, Wasiak S, Guipponi M, Antonarakis SE, Kay BK, Stossel TP, Lamarche-Vane N, and McPherson PS (2001). Endocytic protein intersectin-l regulates actin assembly via Cdc42 and N-WASP. Nat. Cell Biol. 3, 927–932. 10.1038/ncb1001-927. 11584276

[72] Mbefo MK, Fares M-B, Paleologou K, Oueslati A, Yin G, Tenreiro S, Pinto M, Outeiro T, Zweckstetter M, Masliah E, and Lashuel HA (2015). Parkinson disease mutant E46K enhances α-synuclein phosphorylation in mammalian cell lines, in yeast, and in vivo. J. Biol. Chem 290, 9412–9427. 10.1074/jbc.M114.610774. 25657004 PMC4392248

[73] Campoy E, Puig M, Yakymenko I, Lerga-Jaso J, and Cáceres M. (2022). Genomic architecture and functional effects of potential human inversion supergenes. Philos. Trans. R. Soc. Lond. B Biol. Sci 377, 20210209. 10.1098/rstb.2021.0209. 35694745 PMC9189494

