# Supplementary material for: Haploinsufficiency of ITSN1 is associated with a substantial increased risk of Parkinson’s disease
Source: Cell Rep. Author manuscript; Available in PMC 2025 May 30. (PMC12124131; doi:10.1016/j.celrep.2025.115355)
Supplement: 1 [file NIHMS2069108-supplement-1.pdf]

Cell Reports, Volume *44*

Supplemental information

Haploinsufficiency of *ITSN1* is associated with a substantial increased risk of Parkinson's disease

Thomas P. Spargo, Chloe F. Sands, Isabella R. Juan, Jonathan Mitchell, Vida Ravanmehr, Jessica C. Butts, Ruth B. De-Paula, Youngdoo Kim, Fengyuan Hu, Quanli Wang, Dimitrios Vitsios, Manik Garg, Lawrence Middleton, Michal Tyrlik, Mirko Messa, Guillermo del Angel, Daniel G. Calame, Hiba Saade, Laurie Robak, Ben Hollis, Vishnu A. Cuddapah, Huda Y. Zoghbi, Joshua M. Shulman, Slavé Petrovski, Ismael Al-Ramahi, Ioanna Tachmazidou, and Ryan S. Dhindsa

Supplemental Figures

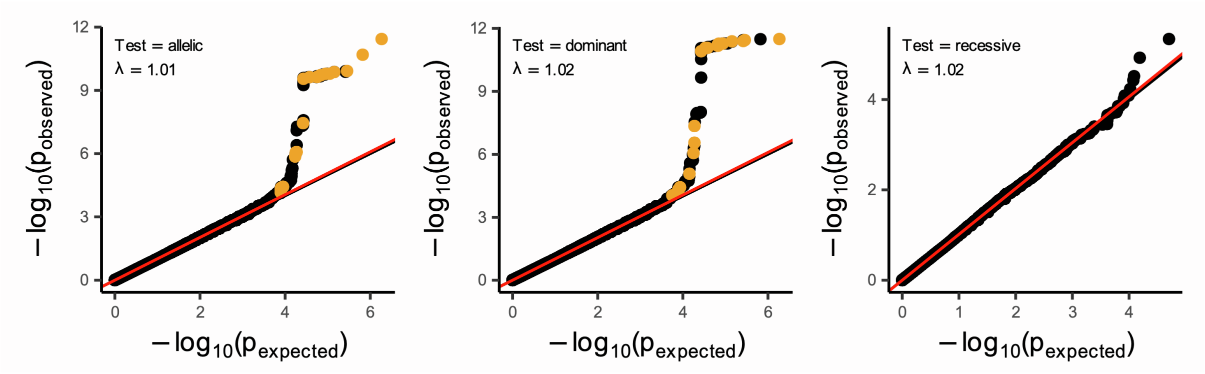

Figure S1. Exome-wide association study QQ plots. Orange points indicate genes that have previously been associated with Parkinson's disease and achieved $p<1x10^{-4}$. The null-distribution of expected p-values is defined based on an n-of-1 permutation of case and control labels. The genomic inflation factor (λ) was calculated using a regression-based approach. Related to Figure 2.

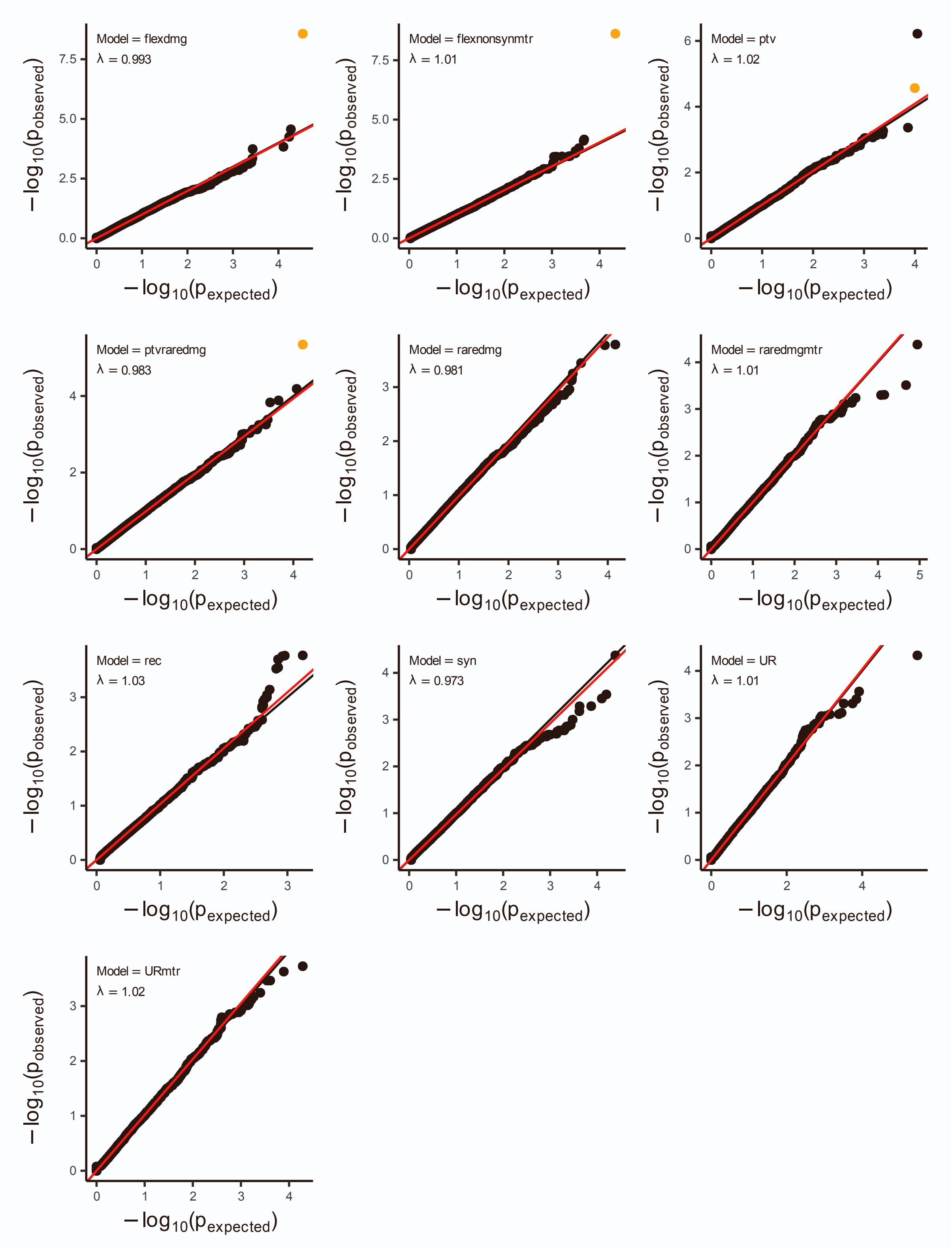

Figure S2. Collapsing analysis QQ plots. Orange points indicate genes that have previously been associated with Parkinson's disease and achieved p<1x10-4. The null-distribution of expected p-values is defined based on an n-of-1 permutation of case and control labels. The genomic inflation factor (λ) was calculated using a regression-based approach. Related to Figure 2.

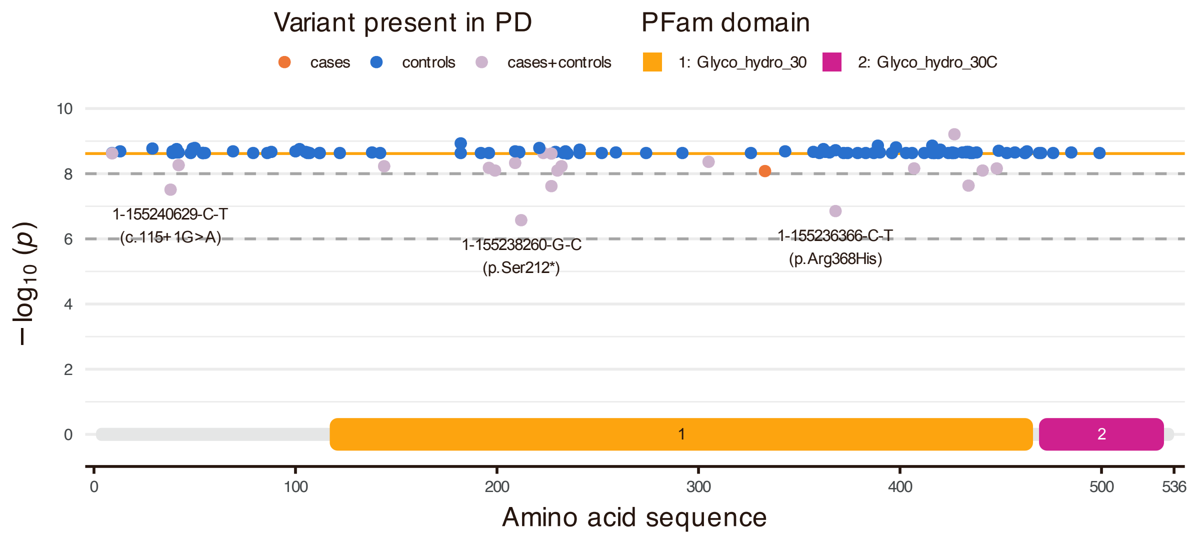

Figure S3. *GBA1* leave-one-out gene-level anlaysis. Two-tailed Fisher's exact tests were performed for the *GBA1* gene under the *flexnonsynmtr c*ollapsing model after removal of a given qualifying variant. x-axis positions of points indicate the location of the removed variant relative to the amino acid sequence of the GBA1 MANE transcript (ENST00000368373) and PFam domains of the corresponding protein (P04062). The orange horizontal line on each panel indicates the association result from the main analysis ($p=2.41x10^{-9}$). Greater deviation from this line indicates greater influence of the removed variant upon the association result. Variants with >1 absolute difference in $-log_{10}(p)$ between the main and leave-one-out analysis results are labelled in the format 'variant ID (MANE transcript consequence: protein impact [or coding sequence impact for splice variants])'. Gray hatched lines indicate significance ($p<1x10^{-8}$) and suggestive ($p<1x10^{-6}$) thresholds. P-values were calculated via a two-tailed Fisher's exact test. Related to Figure 2.

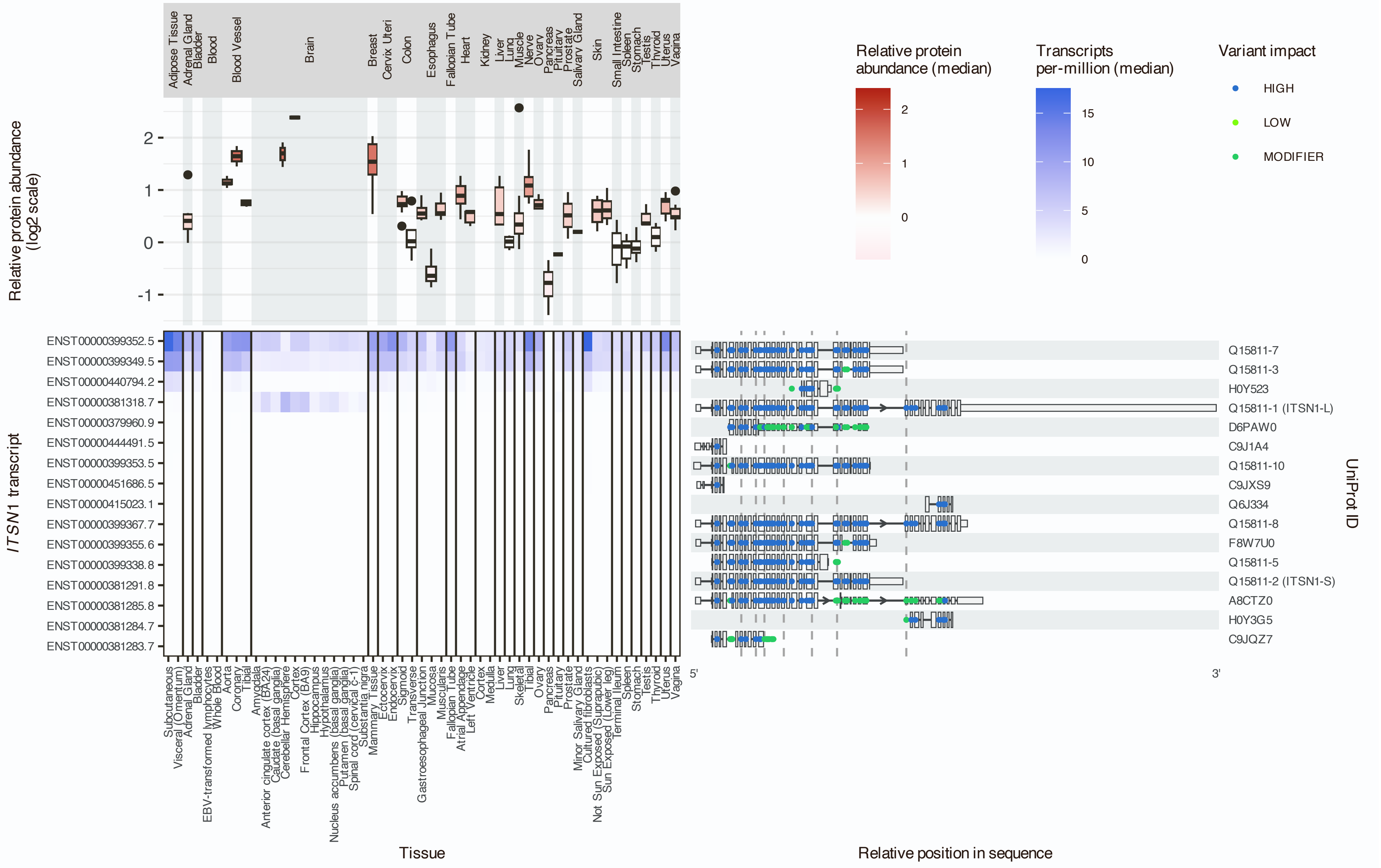

Relative protein abundance (log2 scale)
ITSN1 transcript
Tissue
Relative position in sequence
UniProt ID
Relative protein abundance (median)
Transcripts per-million (median)
Variant impact
HIGH
LOW
MODIFIER
Adipose Tissue
Adrenal Gland
Bladder
Blood
Blood Vessel
Brain
Breast
Cervix Uteri
Colon
Esophagus
Fallopian Tube
Heart
Kidney
Liver
Lung
Muscle
Nerve
Ovary
Pancreas
Pituitary
Prostate
Salivary Gland
Skin
Small Intestine
Spleen
Stomach
Testis
Thyroid
Uterus
Vagina
ENST00000399352.5
ENST00000399349.5
ENST00000440794.2
ENST00000381318.7
ENST00000379960.9
ENST00000444491.5
ENST00000399353.5
ENST00000451686.5
ENST00000415023.1
ENST00000399367.7
ENST00000399355.6
ENST00000399338.8
ENST00000381291.8
ENST00000381285.8
ENST00000381284.7
ENST00000381283.7
Q15811-7
Q15811-3
H0Y523
Q15811-1 (ITSN1-L)
D6PAW0
C9J1A4
Q15811-10
C9JXS9
Q6J334
Q15811-8
F8W7U0
Q15811-5
Q15811-2 (ITSN1-S)
A8CTZ0
H0Y3G5
C9JQZ7
Subcutaneous
Visceral (Omentum)
Adrenal Gland
Bladder
EBV-transformed lymphocytes
Whole Blood
Aorta
Coronary
Tibial
Amygdala
Anterior cingulate cortex (BA24)
Caudate (basal ganglia)
Cerebellar Hemisphere
Cortex
Frontal Cortex (BA9)
Hippocampus
Hypothalamus
Nucleus accumbens (basal ganglia)
Putamen (basal ganglia)
Spinal cord (cervical c-1)
Substantia nigra
Mammary Tissue
Ectocervix
Endocervix
Sigmoid
Transverse
Gastroesophageal Junction
Mucosa
Muscularis
Fallopian Tube
Atrial Appendage
Left Ventricle
Cortex
Medulla
Liver
Lung
Skeletal
Tibial
Ovary
Pancreas
Pituitary
Prostate
Minor Salivary Gland
Cultured fibroblasts
Not Sun Exposed (Suprapubic)
Sun Exposed (Lower leg)
Terminal Ileum
Spleen
Stomach
Testis
Thyroid
Uterus
Vagina
5'
3'

Figure S4. ITSN1 PTVs impact multiple protein-coding transcripts. The heatmap displays expression of different protein-coding ITSN1 transcripts across tissue samples from GTEx, ordered from most to least highly expressed. The panel to the right illustrates the sequence of each transcript and colored points show the location and impact of each ITSN1 variant from the ptv model collapsing analysis against each transcript; HIGH impact indicates a protein-truncating effect; The positions of qualifying PTVs present in people with Parkinson's disease are marked by vertical hatched lines across this panel; intronic sequences that do not overlap exons of another transcript have been shortened to better visualize exons; bars for 5' and 3' untranslated regions are half the height of bars showing the coding sequence. The boxplots above the heatmap display estimates of relative ITSN1 protein abundance across 32 tissue sites in a subset of the GTEx cohort.[1] Related to Figure 2.

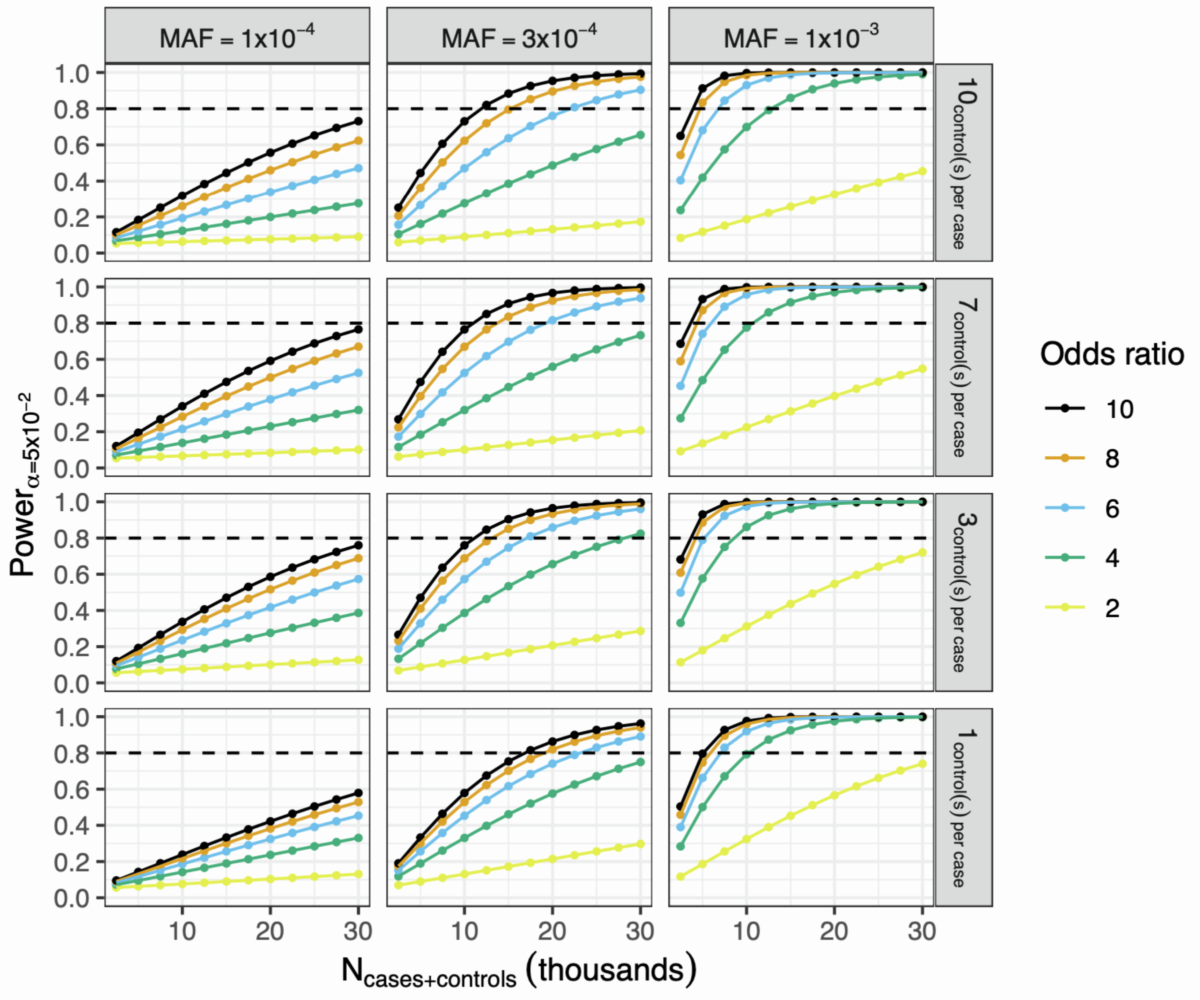

Figure S5. Power analysis of power to detect a dominant genetic association for a rare variant using a dominant test. Points represent the estimated power for a given N (cases+controls) at the effect size (odds ratio) indicated by color. Figure panels are split column-wise according to the minor allele frequency (MAF) for the variant tested (which can be approximately interpreted as the cumulative frequency at which people have a qualifying variant in a gene-level collapsing analysis) and row-wise by the number of controls sampled per case. Analysis was performed with the *genpwr* R package (v1.0.4).[2] Related to STAR methods.

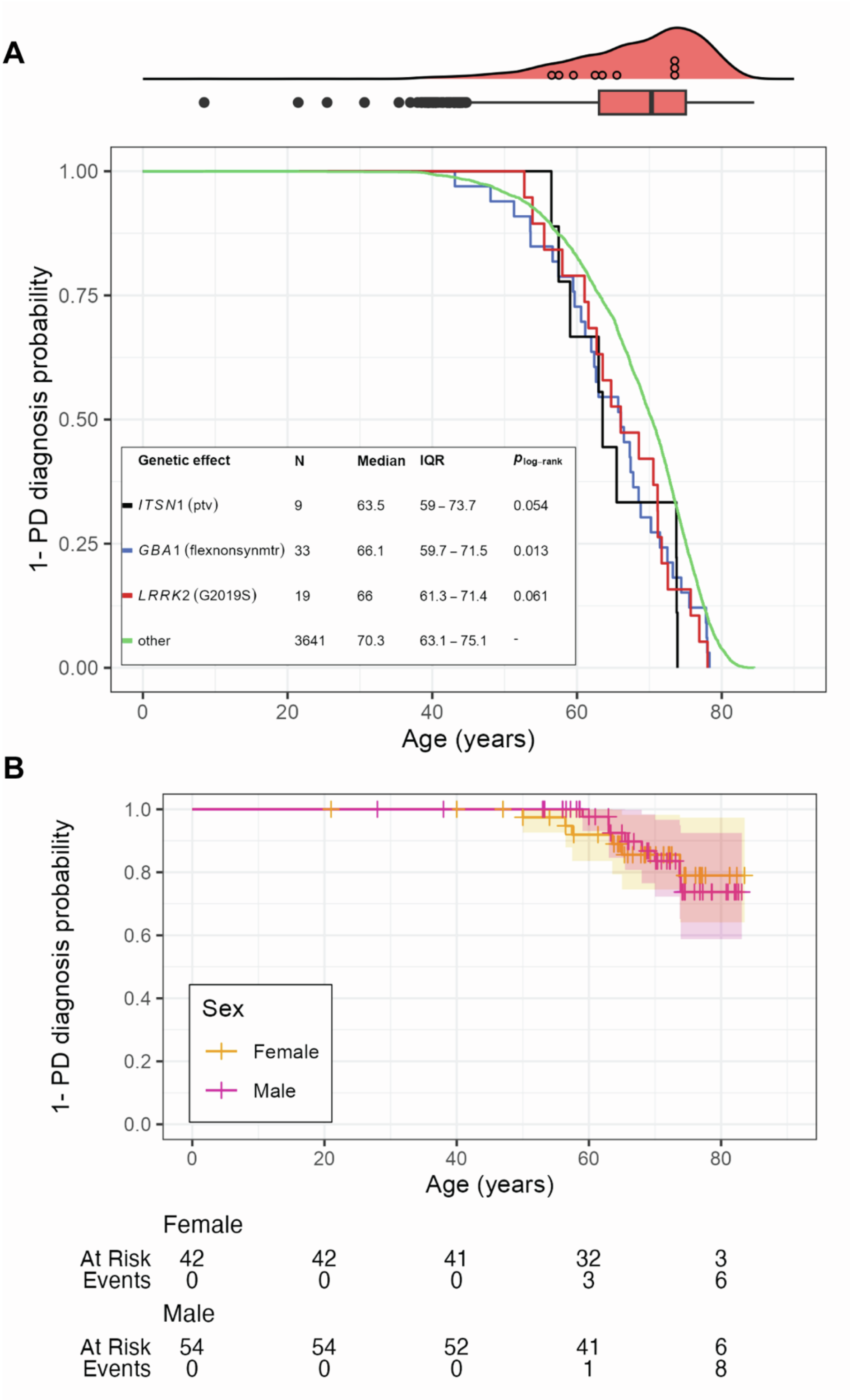

Figure S6. Trends in PD diagnosis age by genetic susceptibility and sex. (A) The top density curve and boxplot show PD diagnosis ages in the UK Biobank European-ancestry cohort; black circles indicate diagnosis ages for individuals with an *ITSN1* PTV, binned by year. The Kaplan-Meier curve depicts time to PD diagnosis from birth, stratified by genetic effect. The *ITSN1* and *GBA1* trend lines include carriers of the most significant gene-level collapsing variants (indicated in parentheses in the figure legend). The *LRRK2* trend line includes carriers of the p.Gly2019Ser variant. The "other" group encompasses all remaining PD cases. The $P_{log\text{-}rank}$ values are from log-rank tests comparing each group against "other." **(B)** Kaplan-Meier curve showing time to PD diagnosis for male and female *ITSN1* PTV carriers in AMP-PD, All of Us, and the 100,000 Genomes Project. Related to Figure 2.

Dataset (reference)	Definition	
	**Cases**	**Controls**
UK Biobank	Presence of ICD-10 G20 code recorded across UK Biobank fields 41270, 41202, 40001, 40002, 40006, 20002, 42031, 42033, and 131023	Absence of any ICD-10 G-chapter code in medical history, indicating diagnosis for a disease of the nervous system (see previous study)[3]
AMP-PD	Labelled as 'Parkinson's disease' or 'Idiopathic PD' by site investigator	Labelled as having no 'No PD Nor Other Neurological disorder' by site investigator
All of Us	N/A	No diagnosis of a mental, behavioral, and neurodevelopmental disorder (F01-F99 ICD-10-CM code) and no diagnosis of a disease of the nervous system (G00-G99 ICD-10-CM code)
100,000 Genomes Project	Recruitment for "Early onset and familial Parkinson's Disease" OR presence of ICD-10 G20 code in hospital records	Not proband or relative of proband recruited for "Neurology and neurodevelopmental disorders"

Table S1. Summary of case and control cohort definitions for genetic association analysis across datasets. Cases from AMP-PD and controls from All of Us and analyzed as a combined case-control cohort. Related to STAR Methods.

Dataset (ancestry group)	N cases (male: female)	N controls (male: female)	N rebalanced controls (male: female)	Odds Ratio [95% CI]	P value	Median age at recruitment (IQR)
UKB (European)	3,702 (2331: 1371)	305,280 (146,949: 158,331)	233,378 (146,949: 86,429)	1.8 [1.7, 2.0]	$1.6x10^{-72}$	58 (50,63)
UKB (South Asian)	69 (43: 26)	7,252 (3,951: 3,301)	NA	1.4 [0.8, 2.3]	0.2	53 (46,60)
UKB (African)	32 (21: 11)	6,528 (2,899: 3,629)	4,418 (2,899: 1519)	2.4 [1.1, 5.5]	0.02	50 (45,57)
UKB (East Asian)	6 (4: 2)	2,053 (704: 1,349)	NA	3.8 [0.5, 42.4]	0.2	52 (45,58)
AMP-PD (European)	3146 (1935: 1211)	NA	NA	NA	NA	65 (58,71)
100kGP (European)	593 (342: 251)	7,402 (5047: 2355)	NA	NA	NA	45 (37,55)
All of Us (European)	NA	50,754 (20,918:29,836)	NA	NA	NA	59 (41,70)

Table S2. Demographic information and sex-rebalancing statistics. Two-sided Fisher's Exact Tests were used to compare the difference in odds of being male versus female across UK Biobank cases and controls. In cohorts with a $p<0.05$, we down sampled the sex overrepresented in controls. The column 'N controls (male: female)' indicates the control cohort sample size before down sampling, and the column 'N rebalanced controls (male: female)' indicates control cohort sample size after down sampling, where performed. UKB = UK Biobank, 100kGP = 100,000 Genomes Project, IQR=Interquartile range. Related to STAR Methods.

Dataset	Genetic ancestry	Carrier frequency in cases	Carrier frequency in controls	N cases with QV	N cases without QV	N controls with QV	N controls without QV
UKB	African	0	0	0	32	0	4418
UKB	East Asian	0	0	0	6	0	2053
UKB	European	2.43×10^{-3}	2.31×10^{-4}	9	3693	54	233324
UKB	South Asian	0	1.38×10^{-4}	0	69	1	7251
AMP-PD	European	1.27×10^{-3}	NA	4	3142	NA	NA
AoU	European	NA	1.77×10^{-4}	NA	NA	9	50745
100kGP	European	1.69×10^{-3}	2.70×10^{-4}	1	592	2	7400
deCODE	European	1.07×10^{-3}	1.01×10^{-4}	5	4663	36	355240

Table S10. Frequencies of *ITSN1* PTVs across cases and controls across different case-control cohorts. AoU = All of Us; 100kGP = 100,000 Genomes Project.

Figure-panel	Genotype
4A	w; GMR-GAL4/+; +
4B	w; GMR-GAL4, UAS-Synulcein/+; +
4C	w; GMR-GAL4, UAS-Synulcein/Dap160Δ1; +
4D	w; GMR-GAL4, UAS-Synulcein/+; UAS-Dap160/+
4F,G-Negative Control	Elav-GAL4(C155)/w; +; +
4F,G-α-Syn/no modifier	Elav-GAL4(C155)/w; UAS-Synuclein/+; +
4F,G-α-Syn/ Dap160(ITSN1)+/-	Elav-GAL4(C155)/w; UAS-Synuclein/Dap160Δ1; +
4F,G-Dap160(ITSN1)+/-	Elav-GAL4(C155)/w; Dap160Δ1/+; +

Table S14. *Drosophila* genotypes.
Complete *Drosophila* genotypes, related to Figure 4.

Note S1

We sought to determine whether suggestive signals from the UKB analysis replicated in the AMP-PD+AoU cohort. Specifically, we tested the genes reported in Table 1 for independent association with PD in the AMP-PD+AoU cohort under their most significant collapsing model. We additionally tested for association to each gene under the *syn* (synonymous) model, which serves as an effective empirical negative control.[3] The synonymous results were non-significant ($p_{FET} \geq 0.05$) in the AMP-PD+AoU cohort, affirming that the dataset is likely to be free from systematic biases (see **Table S11**). We then evaluated combined evidence using exact Cochran-Mantel-Haenszel (CMH) tests by pooling data from the European UKB and AMP-PD+AoU cohorts.

Analyses under the most significant model for each gene showed independent associations significant at $p_{FET}<0.05$ in *GBA1* (*flexnonsynmtr* model)*, ITSN1* (*ptv*)*, and ADH5* (*flexdmg*) (see **Table S11**). The replication of an association to *GBA1 flexnonsynmtr* ($p_{FET} = 7.36 \times 10^{-9}$, OR [95% CI] = 3.76 [2.45, 5.60]; $p_{CMH} = 1.26 \times 10^{-16}$, OR [95% CI] = 3.64 [2.75, 4.73]) offered a robust positive control, given that it is a well-established PD risk gene. Moreover, the AMP-PD dataset is enriched for people with *GBA1*-implicated PD.[4] The *ITSN1* association also replicated under the *ptv* model ($p_{FET} = 5.4 \times 10^{-3}$, OR [95% CI] = 7.18 [1.61, 25.75]; $p_{CMH} = 1.29 \times 10^{-8}$, OR [95% CI] = 9.45 [4.70, 17.74]), providing strong support for *ITSN1* as a PD risk gene. Finally, although *ADH5* showed a modest association with PD, its effect was discordant with the UKB analysis ($p_{FET} = 0.048$, OR [95% CI] = 0.41 [0.13, 0.98]; $p_{CMH} = 0.064$, OR [95% CI] = 1.43 [0.96, 2.05]).

Supplemental References

1 Jiang L, Wang M, Lin S, *et al.* A Quantitative Proteome Map of the Human Body. *Cell* 2020; **183**: 269-283.e19.

2 Moore CM, Jacobson SA, Fingerlin TE. Power and Sample Size Calculations for Genetic Association Studies in the Presence of Genetic Model Misspecification. *Hum Hered* 2020; **84**: 256–71.

3 Wang Q, Dhindsa RS, Carss K, *et al.* Rare variant contribution to human disease in 281,104 UK Biobank exomes. *Nature* 2021; **597**: 527–32.

4 Iwaki H, Leonard HL, Makarious MB, *et al.* Accelerating Medicines Partnership: Parkinson's Disease. Genetic Resource. *Mov Disord* 2021; **36**: 1795–804.
